# USP10 stabilizes FOXM1 through KAT5-mediated acetylation to suppress cell senescence and promote melanoma malignant progression

**DOI:** 10.7150/ijbs.136120

**Published:** 2026-09-10

**Authors:** Zhiyuan Qiu, Chenglian Sun, Juan Wang, Dapeng Liang, Shang Wang, Lili Tian, Tongde Du, Chuanchun Han

**Affiliations:** 1 Institute of Cancer Stem Cell, Dalian Medical University, Dalian, Liaoning 116044, China.; 2 Department of Oncology, The Fourth Affiliated Hospital of Soochow University, Medical Center of Soochow University, Suzhou, Jiangsu 215123, China; 3 Department of Oncology, First Affiliated Hospital of Dalian Medical University, Dalian, Liaoning 116021, China; 4 Jiangsu Cancer Hospital, Jiangsu Institute of Cancer Research, The Affiliated Cancer Hospital of Nanjing Medical University, Nanjing, Jiangsu 210009, China.

**Keywords:** melanoma, USP10, FOXM1, cellular senescence

## Abstract

The ubiquitin signaling cascade plays a pivotal role in multiple cancer types, yet its role in melanoma progression remains incompletely elucidated. An unbiased and systematic analysis indicates that Ubiquitin-specific peptidase 10 (USP10), a member of USP gene family, was top priority prognostic signature for primary and metastatic melanoma progression-free survival and silencing USP10 markedly impairs the growth and metastatic potential of melanoma cells. We further uncover Forkhead box M1 (FOXM1) as a novel substrate of USP10. USP10 directly binds to FOXM1 and removes ubiquitin chains, thereby enhancing FOXM1 protein stability and driving melanoma progression. Notably, the lysine acetyltransferase KAT5 acetylates FOXM1 at residues K422 and K440, which strengthens the interaction between FOXM1 and USP10, facilitating deubiquitination and subsequent stabilization of FOXM1. Additionally, loss of either USP10 or FOXM1 suppresses the expression of the downstream target Telomerase reverse transcriptase (TERT), triggering cellular senescence. Importantly, we identify Golvatinib, as a novel inhibitor of USP10 that effectively curbs melanoma malignancy in both cellular and animal models. Taken together, these findings highlight the pro-tumorigenic role of USP10 in melanoma and suggest that disrupting the USP10/FOXM1 signaling axis could represent a viable therapeutic approach for treating this aggressive cancer.

## Introduction

Melanoma accounts for approximately 90% of deaths from cutaneous cancers 1. Surgical excision is an efficacious treatment for patients with early-stage melanoma 2. However, following complete excision in patients with Stage II-IV melanomas, outcomes can vary due to the rapid metastasis and disease deterioration, with recurrence risk ranging from 30% to 90% 3. Therefore, it is imperative to gain a more profound understanding of the molecular mechanisms of melanoma progression, which will facilitate the identification of new therapeutic targets and the provision of more effective treatment options for melanoma patients.

Ubiquitination, a highly conserved and reversible form of post-translational protein modification, plays a crucial role in cellular protein homeostasis 4, 5. As key participants in the ubiquitination process, deubiquitinating enzymes have the ability to cleave ubiquitin components from ubiquitinated substrates or polyubiquitin chains 6, 7. A substantial body of evidence indicates that deubiquitinating enzymes play a role in a variety of pathological conditions, particularly malignancies 8. For example, USP36 could promote breast cancer progression through ERα signaling, silencing USP36 destabilized the resistant form of ERα (Y537S) and restored sensitivity to tamoxifen 9. Depletion of USP25 sensitized colon cancer cells to IR, 5-Fu, and cisplatin 10. USP5 regulated MDH2 expression through deubiquitination and fostered ripretinib resistance in gastrointestinal stromal tumour 11. In melanoma, USP45 enhance the stability of MRGPRF, which attenuates the PI3K/AKT pathway and functions as a melanoma suppressor 12. USP38 interacts with CTNNB1 to bolster its expression, thereby inhibiting autophagy activation and preventing autophagy-dependent ferroptosis 13. USP22 enhances the transcription of ITGAV/ITGA1/ITGB3, thus facilitating the metastasis of melanoma to distant organs 14.

USP10, a member of the deubiquitinating enzyme family, has been identified as a potential causative agent of human cancers, and its function varies in different types of tumours 15. USP10 exerts its anti-tumour effects by upregulating tumour suppressor proteins, such as p53 in colorectal cancer and PTEN in liver cancer 16, 17. Additionally, it impedes the progression of lung cancer through the activation of the KLF4-TIMP3 pathway 18. Elevated levels of USP10 have been associated with improved survival in patients diagnosed with hepatocellular, lung and colorectal cancer 17-20. In prostate, breast, osteosarcoma and glioblastoma, USP10 has been shown to promote cancer progression through the modulation of immune escape, drug resistance, autophagy and mesenchymal transition 21-24. Despite substantial studies reveal an ambivalent nature for USP10 in the development of tumours, its function in melanoma is still unclear.

In this study, our results uncovered the function of USP10 in melanoma and suggested that USP10 interacted with FOXM1 and maintained its stability. KAT5 targeted FOXM1 at K422 and 440 for acetylation, which enhanced FOXM1 binding affinity to USP10. Furthermore, we also obtained that FOXM1 transcriptionally upregulated TERT expression and suppressed melanoma cell senescence. The small molecule inhibitor Golvatinib was identified as a potential pharmaceutical agent capable of targeting the USP10/FOXM1/TERT axis and inhibited melanoma malignance. In conclusion, the present study provides new avenues for the treatment of melanoma with potential clinical applications.

## Materials and Methods

Details of some assays used in the article were provided in Supplementary Material and Method.

### Cell culture and reagents

A375, A2058, SK-Mel-28 and HEK293T cell lines were obtained from the American Type Culture Collection. These cells were cultured in DMEM (GIBCO-Invitrogen) supplemented with 10% fetal bovine serum (FBS) and in a humid environment of 37°C with 5% CO_2_. The reagents used in this study were listed in Supplementary Table 1.

### Plasmid construction, Lentivirus packaging and infection

The full-length cDNAs of USP10 (wild-type and USP10 inactive mutation C424A) and FOXM1 were cloned into the lentiviral vector pCDH-CMV-MCS-EF1-Puro. The shRNAs targeting USP10 and FOXM1 were constructed into the pLKO.1 vector. Based on the full-length cDNA fragments of USP10, FOXM1and KAT5, we cloned USP10-1 (aa1-99), USP10-2 (aa100-398), and USP10-3 (aa399-798) into the pEGFP-C1 vector; KAT5 (Full-length), KAT5-1 (1-209), KAT5-2 (76-315), KAT5-3 (233-513) into the pEGFP-C1 vector; FOXM1-1 (aa1-235), FOXM1-2 (aa236-347) and FOXM1-3 (aa348-748) were cloned into the pFlag-CMV-2 vector. GST-tagged USP10 were cloned into pGEX-4T-1 vector.

For construction of melanoma cell lines with or without stably expressing or depleting the USP10 or FOXM1, HEK293T cells were co-transfected with the pLKO.1 vector in conjunction with the pVSVG, pREV, pGAG or pCDH vectors and psPAX2, pMD2.G. Following a 48-hour culture period, the cells were subjected to a centrifugation process at 12,000 rpm for a duration of 10 minutes. Thereafter, the viral supernate was collected. Melanoma cells were subsequently infected with the viral solution for a period of 12 hours. Following this, a screening process was conducted after 24 hours, during which 5µg/ml puromycin was used to select stably infected cells. Prior to further analysis, Western blotting was employed to assess infection efficiency in all stably infected cell lines The shRNA sequences and primers for plasmid construction used in this study were listed in Supplementary Table 2. In this study, we used a single shRNA sequence, designated shRNA USP10 #1, to knock down USP10 expression.

For USP10 and FOXM1 KO, USP10 and FOXM1 was knocked out by CRISPR/Cas9: sgRNA design and cloning was performed according to the Feng Zhang laboratory general cloning protocols (Addgene plasmid # 52961; http://n2t.net/addgene:52961; RRID: Addgene 52961). The sgRNAs were cloned into the lentiCRISPR v2 vector (Addgene).

For specific siRNA of TERT were purchased from GenePharma and mixed with Lipofectamine 3000 Transfection Reagent (L3000015, Life Technologies), according to the manufacturer's instructions. Cells were incubated with the transfection complexes for 6 h, followed by incubation in normal medium for 36 h.

### Colony formation and cell migration assays

For colony formation, melanoma cell lines were inoculated into cell 6-well plates (2000 cells per well), and then the cells were placed in a 5% CO_2_ cell culture incubator at 37°C for one week to continue the culture. At the end of the culture, the cells were fixed with 4% paraformaldehyde for 15 minutes and stained with 0.1% crystal violet for 15 minutes. For treatment of melanoma cells with Golvatinib, cells were incubated into cellular 12-well plates (80,000 cells per well), and Golvatinib treatment was added 24 hours later at a working concentration of 2 μM for 48 hours. Subsequently, cells were fixed and stained. The Bio-Rad ChemiDoc XRS system was used for image capture and quantification using ImageJ software.

Cell migration assays were performed by adding 20,000 cells to each Transwell plate, and the migration process was performed on 8 mm polyethylene terephthalate filters (Corning, 3422). After 36 h of incubation in a cell incubator at 5% CO_2_ and 37°C, cells were fixed with 4% paraformaldehyde on the filters for 15 min and then stained with 0.1% crystal violet for 15 min. In the Golvatinib-treated cell migration assay, cells were first treated with Golvatinib (2 μM) for 24 hours. After that, the cells were added to the filter and the drug concentration was maintained for another 36 h. At the end of the treatment, the cells were fixed and stained. Images were acquired using an inverted microscope and then quantified using ImageJ software.

### *In vivo* tumorigenesis and metastasis assay

The animal studies were conducted in accordance with the National Institutes of Health Guide for the Care and Use of Laboratory Animals and received approval from the Animal Research Committee of Dalian Medical University. The nude mice utilized in this study were procured from the Beijing Vital River Laboratory Animal Technology Company and housed in the SPF Laboratory Animal Centre. In the xenograft experiments, 3×10^6^ melanoma cells were resuspended in 100 µl of phosphate buffer and injected subcutaneously into the nude mice. The volume was measured using a vernier caliper every two days, commencing after six days and continuing until the nude mice were euthanised. Subsequently, the xenografts were removed and subjected to analysis. In the xenograft experiments involving Golvatinib, the drug was dissolved in a solution comprising 6% DMSO, 40% PEG300, 5% Tween-80, and 49% saline. This solution was administered by gavage at a concentration of 50 mg/kg on a daily basis. Twelve days later, the mice were euthanised, and the subcutaneous xenograft tumours were excised and subjected to further analysis.

A total of 2×10^6^ A375 cells were resuspended in 100 µl of PBS buffer and injected into the lateral tail vein of nude mice for the lung metastasis assay. The mice were euthanised after 30 days, and the lungs were removed and fixed in 4% paraformaldehyde. The lungs were then embedded in paraffin for subsequent histological analysis.

### Statistical analyses

The analysis was conducted using GraphPad Prism 8.0 software. The data were expressed as either mean ± SD or mean ± SEM. A Student's t-test was employed to ascertain the discrepancies between the experimental and control groups. Correlation analyses were conducted using the Pearson correlation coefficient. P< 0.05 was considered statistically significant.

## Results

### USP gene family member USP10 exert a vital role in melanoma progression

Ubiquitin pathway serves as a crucial regulator, mediating a myriad of cellular events that may contribute to the development and progression of various cancers. Notably, an unbiased and systematic analysis regarding the role of ubiquitin pathway in melanoma progression remains incompletely elucidated. To identify significantly perturbed components of ubiquitin pathway during melanoma progression, we compiled a comprehensive list of Ubiquitin related genes and Deubiquitinating enzyme genes and performed gene set enrichment analysis on RNA-Seq data from The Cancer Genome Atlas melanoma (TCGA-SKCM) cohort in metastatic melanoma patients compared to primary melanoma patients. Our results revealed that USP gene family was the most prominently activated component of ubiquitin pathway (**Fig. 1A**). Moreover, dysfunctional activation of USP gene family was significantly associated with poor progression free survival of primary melanoma patients in TCGA-SKCM cohort (**Fig. 1B**). Particularly, up-regulation of 14 (14/55) USP gene family members were discovered (**Fig. 1C-D**).

Among them, USP10 was top priority prognostic signature for primary and metastatic melanoma progression-free survival (**Fig. 1E-F**). Compared with the normal tissues, USP10 was significantly upregulated in primary melanoma and further increased in metastatic melanoma in a combined cohort of TCGA-SKCM and GTEx-Skin samples (**Fig. 1G**). To verify it, we then analyzed the expression of USP10 in 48 formalin-fixed and paraffin-embedded melanoma tissues and 13 normal tissues using immunohistochemistry (IHC). Consistently, the staining results demonstrated that the expression levels of USP10 were significantly higher in melanoma tissues than in normal tissues and that elevated USP10 expression was positively correlated with lymphatic metastasis in patients with melanoma (**Fig. 1H-I**). Collectively, we hypothesized that elevated expression of USP10 might exert a vital role in melanoma progression.

### USP10 promotes melanoma cell migration and proliferation *in vitro* and *in vivo*

To evaluate the contribution of USP10 to the development and progression of human melanoma, we investigated the effects of USP10 on melanoma cell proliferation and migration *in vitro*. We constructed stable USP10-knockout A375 cells using the CRISPR/Cas9 system, and the knockout efficiency was detected by western blotting (**Fig. 2A**). The colony formation and transwell assays results revealed that loss of USP10 inhibited cell proliferation and migration (**Figs. 2B-E**). To further confirm the function of USP10 on increasing cell proliferation and migration, we knocked down USP10 expression using two independent shRNAs in A375 and A2058 cells and the efficiency of USP10 knockdown was verified by Western blotting (**Supplementary Fig. 1A**). As anticipated, the knockdown of USP10 resulted in a notable reduction in the proliferation and migration capacity of melanoma cells when compared to the control cells (**Figs. 2F-I**). Whereas, melanoma cells with wild-type USP10 overexpression, not the catalytically inactive mutant of USP10 (USP10 C424A), enhanced proliferation and migration (**Figs. 2J-N**).

To further assess the effects of USP10 on melanoma cell metastasis and tumorigenesis *in vivo*, A375 cells with or without USP10 knockout were injected intravenously in nude mice. As shown in **Figs. 2O-Q**, histological analysis revealed that mice receiving USP10-depleted cells had a significant decrease in the number of metastatic lesions compared to control groups. Subsequently, we implanted A375 cells with or without USP10 knockout in nude mice and observed that USP10 depletion notably suppressed the growth of A375 xenograft tumors, as indicated by the reduced tumor weights and tumor sizes compared with those of the control group (**Figs. 2R-T**). Furthermore, the IHC results indicated that A375 xenograft tumor tissues from USP10 depleted cells exhibited a marked reduction in the number of Ki-67 and CD31-positive cells, and an increase in the number of Caspase-3-positive cells (**Supplementary Fig. 1B-C**). Collectively, our data indicate that USP10 promotes malignant progression of melanoma.

### USP10 interacts with FOXM1 and promotes its expression in melanoma cell

To elucidate the underlying molecular mechanism of USP10 promoting melanoma development and progression, we performed integrative analyses of our in-house label-free quantitative proteomics and RNA-Seq data generated from A375 cells with or without USP10 knockout and publicly available RNA-Seq data from TCGA-SKCM cohort stratified by USP10 expression level. For A375 USP10-KO proteomics data, we obtained 131 downregulated and 210 upregulated proteins (**Fig. 3A, Supplementary Fig. 2A and Supplementary Table 3**). Then, we performed the PID (Pathway Interaction Database) pathway enrichment analysis in both A375 USP10-KO proteomics and USP-10 High vs Low TCGA-SKCM RNA-Seq datasets. We identified 4 overlapping pathways that were reversely regulated in the two datasets, including PLK1, FOXM1, AURORA_B and ATR pathway. Particularly, FOXM1 pathway and its master transcriptional regulator FOXM1 were downregulated in A375 USP10-KO proteomics data (**Fig. 3B-D and Supplementary Fig. 2B**).

Meanwhile, 937 downregulated and 1312 upregulated genes were derived from A375 USP10-KO RNA-Seq data (**Supplementary Fig. 2C and Supplementary Table 4**). We further performed PID pathway and transcription factor target enrichment analyses in USP10-KO RNA-seq data. Intriguingly, consistent down-regulation of FOXM1 pathway and its downstream transcriptional targets were reproducibly observed **(Supplementary Fig. 2D-E)**. Moreover, transcriptional targets of FOXM1 were further confirmed to be dramatically elevated in USP-10 High vs Low melanoma patients from TCGA-SKCM cohort (**Supplementary Fig. 2F**). Thereby, multiple lines of evidence across various datasets indicated that FOXM1 might be regulated in a USP10-dependent manner.

To validate our findings, the expression levels of FOXM1 were detected in USP10 knockout or knockdown cells, indicating that loss of USP10 reduced FOXM1 protein levels (**Figs. 3E-F**). Conversely, overexpression of wild-type USP10 increased FOXM1 expression, however, which was abolished by the catalytically inactive mutant of USP10 (C424A), indicating that USP10 promoted FOXM1 expression relying on its deubiquitinating enzymatic activity (**Fig. 3G**). Considering that USP10 usually upregulates its substrates by interacting with them, we thus want to investigate whether USP10 interacted with FOXM1. To this end, we carried out the coimmunoprecipitation (Co-IP) assay to investigate the association between USP10 and FOXM1. The results indicated that ectopically expressed GFP-tagged USP10 could be detected in Flag-tagged FOXM1, and vice versa (**Figs. 3H and Supplementary Fig. 2G**). Furthermore, purified GST-USP10, rather than the GST control group, was able to bind to flag-labeled FOXM1 under cell-free conditions (**Fig. 3I**). More importantly, an interaction between endogenous USP10 and FOXM1 was also detected in melanoma cells (**Fig. 3J**). To map the USP10-binding region on FOXM1, we expressed GFP-tagged USP10 along with Flag-tagged domains of FOXM1 in HEK293T cells. Co-IP assays demonstrated that the DNA binding domain and C-terminal region (aa 235-748) of FOXM was sufficient for its binding with USP10. In addition, the N-terminal region (aa 1-99) of USP10 mediated its physical interaction with FOXM1 (**Figs. 3K-M**). In summary, these results demonstrate that USP10 is a bona fide protein that interacts with FOXM1 and enhances its expression in melanoma cells.

### USP10 maintains FOXM1 stability and abates the ubiquitylation of FOXM1

To further ascertain whether USP10 affects FOXM1 expression in a proteasome-dependent manner, we treated USP10-deficient or sufficient melanoma cell lines with the proteasome inhibitor MG132 and found that MG132 reversed the downregulation of FOXM1 under USP10-deficient conditions (**Figs. 4A-B**). Then, we investigate the influence of USP10 on the stability of FOXM1. Data from cycloheximide chase (CHX) experiments revealed that depletion of USP10 shortened the half-life of FOXM1 (**Figs. 4C-F and Supplementary Figs. 3A-B**). Conversely, overexpression of wild-type USP10, rather than the C424A mutant, enhanced the stability of FOXM1 (**Figs. 4G-H**).

Having verified its effect on the stability of FOXM1, we next assessed the influence of USP10 on the ubiquitylation of FOXM1. As anticipated, loss of USP10 in melanoma cell lines resulted in an increase in FOXM1 polyubiquitination (**Figs. 4I-J**). Furthermore, the ectopic expression of wild-type USP10, not the C424A mutant, was observed to reduce FOXM1 polyubiquitination (**Figs. 4K-L**). To elucidate the specific ubiquitin chain modification of FOXM1 that was removed by USP10, the HA-tagged ubiquitin and its mutants R48K (representing the ubiquitin molecule with only lysine at position 48) and R63K (representing the ubiquitin molecule with only lysine at position 63) were respectively transfected into 293T cells together with USP10 and FOXM1. The results demonstrated that USP10 was capable of removing the K48-linked polyubiquitination of FOXM1 (**Fig. 4M**). Collectively, our data indicate that USP10 elevates the stability of FOXM1 by removing the K48-linked polyubiquitination.

### Acetylation of FOXM1 promotes its binding to USP10 and increases protein stability

Multiple studies have shown that acetylation of oncogenic proteins often increases their stability, thereby augmenting their tumorigenic activity. To explore whether FOXM1 is acetylated in melanoma, A375 and A2058 cells were treated with trichostatin A (TSA), an inhibitor of class I/II histone deacetylases (HDACs), or nicotinamide (NAM), an inhibitor of sirtuin (SIRT) family deacetylases. Our results revealed that NAM markedly increased FOXM1 protein levels, whereas TSA reduced FOXM1 expression-findings consistent with previous reports 25, 26 (**Supplementary Figs. 4A-C**). Further analysis using cycloheximide (CHX) chase and ubiquitination assays indicated that NAM significantly prolonged the half-life of FOXM1 and suppressed its ubiquitination (**Supplementary Figs. 4D-F**). Next, we examined endogenous FOXM1 acetylation using a pan-acetylation antibody. Immunoblotting confirmed that FOXM1 undergoes acetylation, and this modification was substantially enhanced upon NAM treatment** (Figs. 5A-B)**. Since lysine residues are common targets for acetylation, we analyzed available proteomic data (https://www.phosphosite.org/) and identified five potential acetylation sites: Lys63, Lys422, Lys440, Lys603, and Lys614 (**Supplementary Fig. 4G**). Site-directed mutagenesis substituting each lysine with arginine revealed that K422 and K440 are the major acetylation sites on FOXM1 (**Fig. 5C**).To assess how acetylation at K422 and K440 influences FOXM1 stability, wild-type (WT) FOXM1, acetylation-deficient mutants (single site mutant K422R and K440R, a double site mutant K422/440R), and acetylation-mimetic mutants (single site mutant K422Q and K440Q, a double site mutant K422/440Q) were expressed in 293T cells. CHX chase experiments showed that the K422R, K440R and K422/440R mutants had shorter protein half-lives compared to WT FOXM1, while the K422Q, K440Q and K422/440Q mutants exhibited greater stability (**Figs. 5D-E and Supplementary Fig. 4H-I**). These results indicate that acetylation at K422 and K440 plays a critical role in regulating FOXM1 protein turnover.

We further explored the relationship between FOXM1 acetylation and USP10-mediated regulation of FOXM1 expression. Knockdown of USP10 abolished the NAM-induced increase in FOXM1 protein levels and stability (**Figs. 5F-H and Supplementary Figs. 4J-L**). Co-immunoprecipitation assays demonstrated that enhanced FOXM1 acetylation-either through NAM treatment or expression of the K422Q, K440Q or K422/440Q mutants-strengthened its interaction with USP10 (**Figs. 5I-J**). In contrast, the K422R, K440R and K422/440R mutants displayed weakened binding to USP10 (**Fig. 5K**). In summary, these findings demonstrate that K422 and K440 are key acetylation sites on FOXM1, and that acetylation at these residues promotes USP10 binding, leading to increased protein stability.

### FOXM1 is acetylated by KAT5 at lysine 422 and 440

Acetylation represents a reversible post-translational modification that is tightly controlled by acetyltransferases and deacetylases. Mass spectrometry analysis identified several interacting partners of FOXM1, including the histone acetyltransferase KAT5 (**Supplementary Figs. 5A and Supplementary Table 5**). We confirmed the physical association between endogenous FOXM1 and KAT5 in melanoma cells (**Fig. 6A**). Additionally, co-expression experiments showed that GFP-tagged KAT5 co-precipitated with Flag-tagged FOXM1, and reciprocal pulldown validated their interaction (**Figs. 6B-C**).To map the specific domains responsible for the FOXM1-KAT5 interaction, deletion mutants of Flag-FOXM1 and GFP-KAT5 were generated and subjected to co-immunoprecipitation (Co-IP) assays. These analyses revealed that KAT5 binds to the DNA-binding domain (DBD) of FOXM1, whereas FOXM1 primarily interacts with the zinc finger (Zn) domain of KAT5 (**Figs. 6D-F**).

Next, we examined the influence of KAT5 on FOXM1 acetylation. We observed that overexpression of KAT5 enhanced FOXM1 acetylation in melanoma cells, and conversely, depletion of KAT5 reduced this modification (**Figs. 6G-H and Supplementary Figs. 5B-C**). However, acetylation-deficient mutants (a double site mutant K422/440R) abolished the KAT5-enhanced acetylation of FOXM1 (**Fig. 6I**). Taken together, these results demonstrate that KAT5 directly interacts with FOXM1 and mediates its acetylation at K422 and K440 sites.

### KAT5 suppresses FOXM1 degradation and enhances its binding to USP10 through regulating FOXM1 acetylation

To assess the functional significance of KAT5 in regulating FOXM1 protein levels, we silenced KAT5 in A375 and A2058 cells and monitored FOXM1 expression. We obtained that KAT5 knockdown led to a marked decrease in FOXM1 protein abundance (**Figs. 7A-B**). Loss of KAT5 shortened the half-life of FOXM1 and increased its ubiquitination relative to control cells (**Figs. 7C-H**).

Furthermore, elevated KAT5 levels strengthened the interaction between FOXM1 and USP10, while KAT5 silencing impaired their interaction (**Fig. 7I-L**). Taken together, these results demonstrate that KAT5 mediates FOXM1 acetylation, which in turn facilitates the recruitment of USP10, ultimately contributing to the stabilization of FOXM1.

### USP10 facilitates melanoma progression by upregulating FOXM1

To investigate whether USP10 facilitates melanoma progression by upregulating FOXM1, we thus overexpressed FOXM1 in USP10-depleted melanoma cells and verified the expression levels of USP10 and FOXM1 by western blotting (**Fig. 8A**). Then, we evaluated the alteration of cell migration by Transwell assay and found that USP10 knockdown dramatically suppressed cell migration, however the inhibition was reversed when FOXM1 was overexpressed (**Figs. 8B-C**). Consistently, the results from lung metastasis xenograft model indicated that a reduction of the number of lung nodules in USP10 depleted cells was recovered by FOXM1 overexpression (**Figs. 8D-F**).

Furthermore, we also assessed the effect on cell proliferation by colony formation assay. Similarly, increased FOXM1 expression abolished the reduction in cell proliferation by USP10 knockdown (**Figs. 8G-H**). To further confirm the functional consequence of FOXM1 overexpression on the tumor-promoting role of USP10, the xenograft tumour formation assay was performed. We observed that overexpression of FOXM1 abrogated the inhibitory influence of USP10 depletion on tumorigenesis (**Fig. 8I-K**). To further confirm this point, we overexpressed USP10 in FOXM1-depleted melanoma cells and examined the corresponding changes in cell migration and proliferation. As shown in **Supplementary Figs. 6A-E**, USP10 overexpression did not reverse the inhibitory effects of FOXM1 knockdown on cell migration and proliferation.

Finally, to better validate the clinical relevance of USP10/FOXM1 axis in melanoma, IHC staining and correlation analysis were performed to investigate the association between USP10 and FOXM1. The results revealed that increased FOXM1 was positively associated with USP10 high expression in melanoma (**Figs. 8L-M**)**.** In summary, our data indicate that USP10 promotes melanoma progression via the upregulation of FOXM1 expression in melanoma.

### FOXM1 transcriptionally upregulates TERT in melanoma cells

To illustrate the potential molecular mechanisms by which USP10/FOXM1 axis promotes melanoma development and progression, RNA-seq was performed to identify differentially expressed genes (USP10-KO DEGs and FOXM1-KO DEGs) in USP10- and FOXM1-deficient A375 cells respectively. In addition, we uncovered FOXM1-coexpressed genes from RNA-Seq data in TCGA-SKCM cohort. Then, we screened the potential USP10/FOXM1 axis downstream targets by comparing the USP10-KO DEGs, FOXM1-KO DEGs and FOXM1-coexpressed genes. 15 genes were overlapped among USP10-KO Up-DEGs, FOXM1-KO Up-DEGs and FOXM1 negatively co-expressed genes. 12 genes were overlapped among USP10-KO Down-DEGs, FOXM1-KO Down-DEGs and FOXM1 positively co-expressed genes. Among these 27 overlapping genes, TERT was regarded as top priority candidate target gene with the most pronounced changes (**Fig. 9A-C**). We thus selected TERT for further validation. Consistently with the RNA-seq data, depletion of USP10 and FOXM1 significantly decreased TERT expression in melanoma cells (**Figs. 8D-G and Supplementary Figs. 7A-B**). The overexpression of USP10 and FOXM1 significantly increased the expression of TERT (**Supplementary Figs. 7C-D**). Meanwhile, the reduction of TERT induced by USP10 knockout could be reversed by FOXM1 overexpression (**Fig. 9H**).

To validate whether FOXM1 transcriptionally upregulated TERT expression, we utilized the JASPAR database (https://jaspar.genereg.net) to analyze the sequence of TERT promoter and identify a potential binding site of FOXM1 on TERT promoter.

We then inserted the promoter region of TERT containing the wild-type binding site (WT) of FOXM1 or the mutant binding site (Mut) into pGL3**-**based luciferase reporter vectors (**Fig. 9I**). Subsequently, the two plasmids were transfected into 293T cells with or without FOXM1 overexpression, respectively. The results revealed that the luciferase activity of WT not the Mut was significantly increased in FOXM1 overexpressing cells (**Fig. 9J**). Whereas, depletion of FOXM1 or USP10 reduced the luciferase activity of WT (**Figs. 9K-L**).

Furthermore, the ChIP assays revealed that chromatin fragments containing the wild-type binding site were preferentially enriched in anti**-**FOXM1 immunoprecipitants relative to IgG controls. The binding affinity of FOXM1 for the TERT promoter was diminished in FOXM1-deficient cells (**Fig. 9M**). Taken together, these results indicate that FOXM1 directly binds to the promoter of TERT and transcriptionally upregulates its expression.

### Deficiency of USP10 and FOXM1 triggers melanoma cell senescence

Previous studies indicate that TERT represents a pivotal component of the telomerase, which exerts a profound influence on the pathogenesis of cancer by maintaining telomere length and enabling cells to evade cellular senescence 27. Therefore, we suspected that inhibition of USP10/FOXM1 axis might promote cellular senescence. We collected two human tissue ubiquitous cellular senescence gene sets, including SenMayo and CoreScence, and a human skin-specific cellular senescence gene set SenSkin. We then performed senescence gene set enrichment analysis in USP10- and FOXM1-deficient A375 cells. The results revealed that all the senescence gene sets were remarkably activated in both USP10- and FOXM1-deficient A375 cells (**Supplementary Figs. 8A-B**). Furthermore, we performed senescence gene set enrichment analysis in USP-10 High vs Low and FOXM1 High vs Low melanoma patients in TCGA-SKCM cohort. Consistently, all the senescence gene sets were remarkably suppressed in both USP-10 High vs Low and FOXM1 High vs Low melanoma patients (**Supplementary Figs. 8C-D**). To verify the impact of USP10 and FOXM1 on melanoma cell senescence, β-Gal staining was performed to assess the cell senescence in USP10 and FOXM1 depleting melanoma cells. We observed that loss of USP10 and FOXM1 elevated the proportion of β-Gal-positive cells (**Figs. 10A-D and Supplementary Figs. 9A-D**), and the mRNA levels of numerous SASP factors (including IL-1α, IL-1β, IL-12α, TNFα, and CXCL8) (**Figs. 10E-F**). In addition, inhibition of USP10 and FOXM1 also resulted in the senescence-related genes P21 and P27 upregulation, accompanying CDK4 and CDK6 downregulation (**Figs. 10G-H and Supplementary Figs. 9E-F**). These results indicated that the loss of USP10 and FOXM1 induced melanoma cell senescence.

Then we investigated whether the influence of USP10/FOXM1 axis on cell senescence in a TERT dependent manner. To this end, we knocked down TERT in FOXM1 overexpressing melanoma cells and found that silence of TERT abolished the suppressive influences of FOXM1 on cell senescence (**Figs. 10I-K and Supplementary Figs. 9G-I**). Meanwhile, our findings also demonstrated that the silence of TERT weaken promoting effects of FOXM1 on cell migration and proliferation (**Figs. 10L-O and Supplementary Figs. 9J-M**). In summary, our data suggest that USP10/FOXM1 axis suppressed melanoma cell senescence and promoted malignant progression by upregulating TERT expression.

### Golvatinib binds with USP10 to suppress the USP10/FOXM1 axis

Given the important role of USP10 in elevating FOXM1 stability and promoting melanoma progression, we reasoned that targeting USP10 may be beneficial in the treatment of melanoma. To prove this hypothesis, we first predicted the crystal full-length structure of the USP10 by D-I-TASSER. Based on this structure, the catalytic pockets of USP10 are predicted by the COACH (**Fig. 11A**). We then utilized virtual screening to identify small molecules coming from FDA-approved drugs library that bind the catalytic pockets of USP10. According to their simulated binding energy, we selected the top 10 compounds with a docking energy ≤-10.0 kcal/mol (**Figs. 11B-C**) and assessed their effects on the expression of USP10 and FOXM1. Among the 10 compounds, Golvatinib and Bemcentinib displayed a high inhibitory ability for USP10 and FOXM1 expression in both A375 and A2058 cells (**Supplementary Figs. 10A-B**). Interestingly, the further results showed that the downregulation of FOXM1 induced by Golvatinib not Bemcentinib was disappeared in USP10-deficient cells demonstrating the suppressive effect of Golvatinib on FOXM1was relying on USP10 (**Figs. 11D-F and Supplementary Figs. 10C-E**). To further verify it, we treated melanoma cells with different dose of Golvatinib and found that Golvatinib significantly suppressed the expression of USP10/FOXM1axis and their downstream gene TERT (**Fig. 11G**). Meanwhile, Golvatinib reduced the stability of FOXM1 and increased ubiquitinated FOXM1 levels in a dose-dependent manner (**Figs. 11H-J and Supplementary Figs. 10F-H**). Then, the *in vitro* ubiquitination assay demonstrated that Golvatinib abolished the deubiquitination effect of USP10 on FOXM1 (**Supplementary Figs. 10I**).

Subsequently, the Cellular Thermal Shift Assay (CETSA) was performed to verify the interaction of Golvatinib to USP10. A375 cells treated with or without Golvatinib were subjected to CETSA heat pulse. As expected, the heat challenge quickly resulted in USP10 denaturation and precipitation while Golvatinib dramatically elevated the thermal stability of USP10 protein at higher temperatures, indicating a direct binding of Golvatinib to USP10 (**Figs. 11K-L**). Furthermore, the predicted protein-ligand interactions model showed that Golvatinib formed multiple interactions with USP10, including hydrophobic interactions with Ile610, Gln626, Phe628, Phe629, and Val673, hydrogen bonds with Asn533, salt bridges with Asp523 and Glu525, and halogen bonds with Lys687 (**Figs. 11M-N**). Taken together, these data demonstrate that Golvatinib is a small molecular inhibitor of USP10 and suppresses activation of USP10/ FOXM1axis.

### Golvatinib inhibits malignant progression of melanoma by targeting USP10

Golvatinib, which is the first kinase inhibitor with dual action against both c-Met and VEGFR-2 28. Its role in malignant tumours and the underlying mechanisms remain elusive. To evaluate the therapeutic potential of Golvatinib in melanoma, A375 and A2058 cells were treated with 0, 2 and 5μM Golvatinib. We observed that Golvatinib significantly reduced cell migration and proliferation, as well as increasing cell senescence (**Figs. 12A-F and Supplementary Figs. 11A-C**).

Subsequently, we investigated the inhibitory influence of Golvatinib on tumor metastasis and growth *in vivo*. As shown in **Figs. 12G-I**, the tail vein metastasis trial revealed that mice receiving Golvatinib treatment had a significant decrease in the number of metastatic lesions than the control group. Then, the tumor formation assay indicated that Golvatinib substantially suppressed the growth of A375 xenograft tumors, as indicated by the reduced tumor weights and tumor sizes compared with those of the control group (**Figs. 12J-L**). The subsequent western blotting and IHC results indicated that A375 xenograft tumor tissues from Golvatinib treatment group showed a significant reduction in USP10, FOXM1 and TERT expression as well as a decrease in the number of Ki-67 and CD31-positive cells, and an increase in the number of Caspase-3-positive cells (**Figs. 12M-O**).

To better verify that the inhibitory role of Golvatinib in melanoma cell was relied on USP10, we also assessed the influence of Golvatinib on cell migration and growth in melanoma cells with or without USP10 depletion. We found that loss of USP10 abolished the suppressive role of Golvatinib in melanoma cells (**Supplementary Figs. 12A-H**). Consistently, Golvatinib had no obvious inhibitory effect on tumor formation of USP10 KO A375 cells *in vivo* (**Supplementary Figs. 12I-K**). Taken together, these data suggest that Golvatinib exhibits therapeutic potential in melanoma by targeting USP10.

## Discussion

In this study, we described the oncogenic role of USP10 in melanoma for the first time. USP10 interacted with FOXM1 and suppressed its degradation. KAT5 increases acetylation of FOXM1 and further enhances its interaction with USP10. Increased FOXM1 bound to the promoter of TERT and facilitated its expression, thereby, suppressing melanoma cell senescence and enhancing cell proliferation and metastasis. In addition, we also verified that Golvatinib was a new USP10 inhibitor and inhibited melanoma malignant progression by targeting the USP10/FOXM1 axis.

USP10 has been shown to exist different functions during tumourigenesis 15. For instance, elevated USP10 expression impeded KLF4 degradation and inhibited lung cancer development 18. Depletion of USP10 hindered LKB1 activity and contributed to HCC formation 29. In contrast, USP10 accelerated osteosarcoma progression by stabilizing GSK3β 23. In breast cancer, USP10 facilitated cell metastasis by promoting the stability of IGF2BP1 and TCF4 30, 31. Similarly, we demonstrated that USP10 was highly expressed in melanoma tissues and promoted cell proliferation and metastasis. Then, the label-free quantitative proteomics was employed to identify the downstream target proteins of USP10 and showed that USP10 deficiency significantly downregulated FOXM1 expression.

Forkhead box M1 (FOXM1) is an evolutionarily conserved member of the forkhead box family of transcription factors, exhibits predominant activity in tissues with high proliferative capacity, including both normal and neoplastic cells 32. FOXM1 participates in numerous physiological and pathophysiological processes in humans, encompassing embryonic development, tissue regeneration, chemoresistance, and oncogenesis 33-36. FOXM1 was reported to be involved in medicating progression of a variety of human cancers, such as prostate cancer 37, breast cancer 38, melanoma 39 and lung cancer 40. Therefore, the inhibition of FOXM1 could emerge as a promising therapeutic strategy for cancer treatment. The development of FOXM1 inhibitors is frequently regarded as challenging due to the complexity inherent in identifying the drug binding regions of transcription factors, which is further compounded by the extensive interaction surfaces 41. Encouragingly, FOXM1 is unstably protein and can be modified by ubiquitination 42. Thus, influencing ubiquitination modification processes can also achieve the purpose of FOXM1 inhibition. Our data demonstrated that USP10 functions as a deubiquitinating enzyme, thereby stabilizing FOXM1 protein levels through deubiquitination. Furthermore, the restoration of FOXM1 in cells exhibiting USP10 knockdown effectively counteracted the inhibition of proliferation and metastasis caused by USP10 depletion.

In addition to ubiquitination, the FOXM1 protein undergoes various PTMs, including phosphorylation, SUMOylation, acetylation, and methylation, which may exert either activating or inhibitory effects. The transcriptional activity of FOXM1 is upregulated during the cell cycle, correlating with its phosphorylation levels 43. Multiple sites on FOXM1 are modified by SUMO-2, which inhibits FOXM1 dimerization and diminishes its self-inhibition, consequently enhancing its transcriptional activity 44. The SUMOylation of FOXM1 at K463 by SUMO-1 is also critical for its transcriptional function 45. Furthermore, FOXM1 is regulated by methyltransferases and acetyltransferases. Under normoxic conditions, the methyltransferase SETD3 specifically binds to and methylates FOXM1, thereby inhibiting its activity 46. Acetylation of FOXM1 by p300/CBP enhances its transcriptional activity by increasing its DNA binding affinity, protein stability, and sensitivity to phosphorylation 47. In this study, we identified KAT5 as a novel acetyltransferase of FOXM1, which acetylates FOXM1 at K422 and K440, thereby enhancing its interaction with USP10 and promoting melanoma progression. Additionally, we also investigated the tumor promotion effects of KAT5 by FOXM1 acetylation. We found that knockdown of KAT5 inhibits proliferation, metastasis, and induces cellular senescence. Moreover, overexpression of FOXM1 reverses the reduction in melanoma malignancy caused by KAT5 knockdown indicating that FOXM1 is a key downstream regulatory protein of KAT5 (**Supplementary Figs. 13**).

Given FOXM1's oncogenic properties, increased studies have focused on its transcriptional regulation of downstream gene targets. Consistently, our findings demonstrated that USP10 and FOXM1 cooperatively modulated TERT gene expression. FOXM1 directly bound to the TERT promoter and increased TERT expression. TERT is a crucial component of telomerase, and its primary influence on cancer formation is through maintenance of telomere length and enabling cells to evade cellular senescence 48, 49. Our findings indicated that the absence of USP10 and FOXM1 led to a notable elevation in the incidence of cellular senescence in melanoma cells. This observation suggested that the promotion of melanoma progression by the USP10/FOXM1 pathway may be attributed to the upregulation of TERT, which enabled cells to evade the process of cellular senescence.

Currently, the ubiquitination process has emerged as a potential drug target for anti-cancer therapeutic strategies 5, 50. USP10 has been reported to play critical roles in various cancers, the screening and development of USP10 inhibitors holds significant promise in cancer treatment. However, only a small number of USP10 inhibitors have been identified for cancer treatment. For example, Compound D1 containing the quinoline-4(1H)-1 scaffold was identified as a novel USP10 inhibitor, which inhibited USP10 to promote YAP degradation and ultimately triggered s-phase blockade in HCC cells 51. Spautin-1, has been reported as a USP10 and USP13 antagonist and exhibited synergy with cisplatin in the treatment of melanoma 52. Wu-5, another USP10 inhibitor, enhanced crenolanib-induced AML cell death by inhibiting FLT3 and AMPK pathways 53. Nevertheless, the anticancer efficacy and specificity of compound D1 *in vivo* have yet to be demonstrated 51, Spautin-1 has not been reported to bind directly to USP10, which has led to uncertainty as to its specificity 52. Wu-5 showed potential efficacy against acute myeloid leukemia, its clinical toxicity risk remains uncertain 53. Therefore, the identification and development of new specific inhibitors of USP10 continues to be of considerable value for cancer therapy.

At present, repurposing existing drugs to treat new indications is a new drug development strategy. The compounds in the FDA-approved Drug Library have clear biological activity and safety data, thus rendering it as an important tool in the processes of drug screening and development. A library of FDA-approved drugs was screened to identify potential inhibitors of USP10, Golvatinib, a dual inhibitor of c-Met and VEGFR-2 tyrosine kinases, was identified as a result of this process. Our data suggested that the ability of the drug to inhibit USP10 expression in melanoma cells and to reduce FOXM1 protein levels via USP10. Additionally, Golvatinib effectively inhibited melanoma cells *in vitro* and* in vivo* and promoted cellular senescence. The inhibitory effect of Golvatinib was weakened in USP10-deficient conditions, suggesting that Golvatinib exerted its therapeutic effect in melanoma by targeting USP10 and then affecting downstream FOXM1.

However, our study has several limitations: Firstly, the reasons underlying the elevated expression of USP10 in melanoma remain unclear, as do the potential transcriptional regulatory mechanisms or epigenetic modifications that may govern USP10 expression. Secondly, in melanoma, both USP10 and FOXM1 are highly expressed, and USP10 promotes the stability of FOXM1. However, in some other types of tumors, the expression of USP10 and FOXM1 is inconsistent: USP10 is lowly expressed while FOXM1 is highly expressed. Whether USP10 can regulate FOXM1 still requires further investigation.

## Conclusions

In summary, our findings established that USP10 fostered melanoma progression by the stabilization of FOXM1. Furthermore, Golvatinib, a novel inhibitor of USP10, was found to disrupt the USP10/FOXM1 signaling axis, thereby suppressing melanoma cell proliferation and metastasis and triggering cellular senescence. These discoveries could pave the way for novel therapeutic strategies in the clinical management of melanoma.

## Supplementary Material

Supplementary figures.

Supplementary materials and methods.

Supplementary table 1.

Supplementary table 2.

Supplementary table 3: SP10KO vs A375WT Proteomics DEPs.

Supplementary table 4: USP10KO vs A375WT rnaseq DEGs.

Supplementary table 5: Data of FOXM1IP mass.

## Figures and Tables

**Figure 1:**
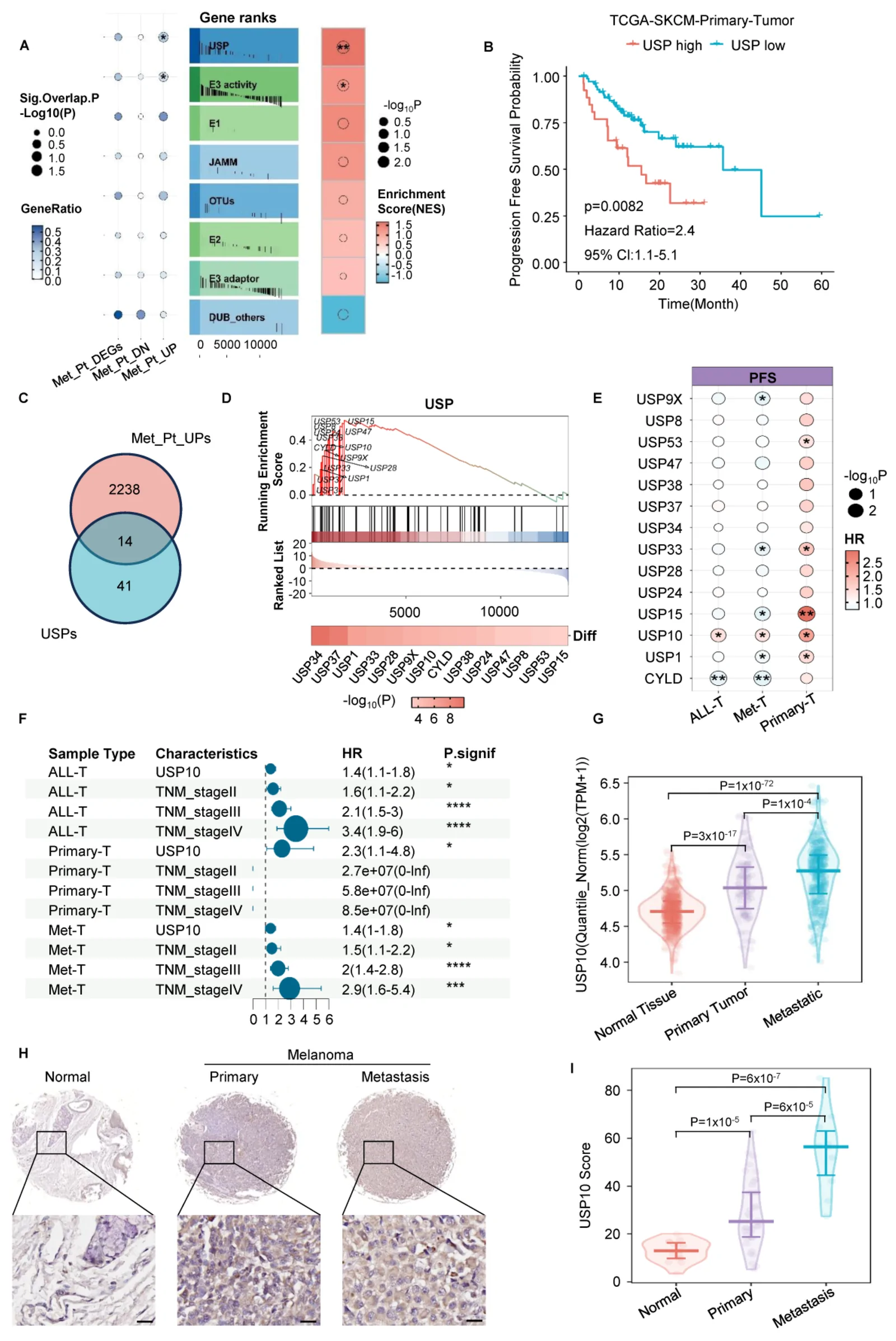
** USP10 is top priority member associated with adverse progression-free survival in melanoma.** (A) Ubiqutin pathway related gene sets enrichment analysis in metastatic versus primary melanoma from TCGA-SKCM cohort. Dotplot (Left) showing enrichment statistics calculated by hypergeometric tests. The color bar denotes proportion of significantly up and down-regulated genes mapping to the corresponding ubiquitin pathway related genesets respectively. Circle size represents enrichment significance -log10 (P value). Dotplot (Right) showing enrichment statistics calculated by GSEA. The color bar denotes normalized enrichment score (NES). Circle size represents the enrichment significance -log10 (P value). Met_Pt_DEGs: metastatic versus primary melanoma different expression genes; Met_Pt_UP : metastatic versus primary melanoma upregulated genes; Met_Pt_DN metastatic versus primary melanoma downregulated genes. (B) Kaplan-Meier survival curves showing significantly worse progression free survival in USP-high versus USP-low melanoma patients from TCGA-SKCM cohort. Two-sided log-rank test. (C-D) Venn diagram showing overlapping genes between USP gene family members and up-regulated genes in metastatic versus primary melanoma from TCGA-SKCM cohort. Gene set enrichment analysis (GSEA) of USP gene family in metastatic versus primary melanoma from TCGA-SKCM cohort. Significantly up-regulated USP gene family members were highlighted and labeled. The color bar denotes differential expression significance -log10 (P value). (E-F) Multivariate Cox regression analysis for USP gene family. USP10 expression was the top significant risk factor for PFS in both primary and metastatic melanoma patients from TCGA-SKCM cohort after adjusting for TNM stage. The color bar denotes hazard ratio (HR) and circle size represents the significance -log10 (P value) of individual USP gene family member in the multivariate Cox model. Forest plot of the multivariate Cox regression analysis showing hazard ratio estimates and 95% confidence intervals from USP10 expression level and TNM stage (stage I defined as reference level) in TCGA-SKCM cohort. Statistical significance among levels for each variable was determined by two-sided Wald test. ALL-T: All-Tumor; Met-T: Metastatic-Tumor; Primary-T: Primary-Tumor. (G)Violin plot of USP10 expression level in normal tissue, primary melanoma and metastatic melanoma in a combined cohort of TCGA-SKCM and GTEx-Skin samples. (H-I) Immunohistochemical staining for USP10 was conducted on normal tissues (n=13), primary melanoma tissues (n=38), and metastatic melanoma tissues(n=10), followed by an assessment of USP10 expression. Scale bars: 20 μm. Violinplot of USP10 expression level in normal tissue, primary and metastatic melanoma tissue. G, I data analyzed by Non-parametric Mann-Whitney test. For A, E and F data, P value< 0.05 are marked with asterisks, *P < 0.05, **P < 0.01, ***P < 0.001, ****P<0.0001.

**Figure 2:**
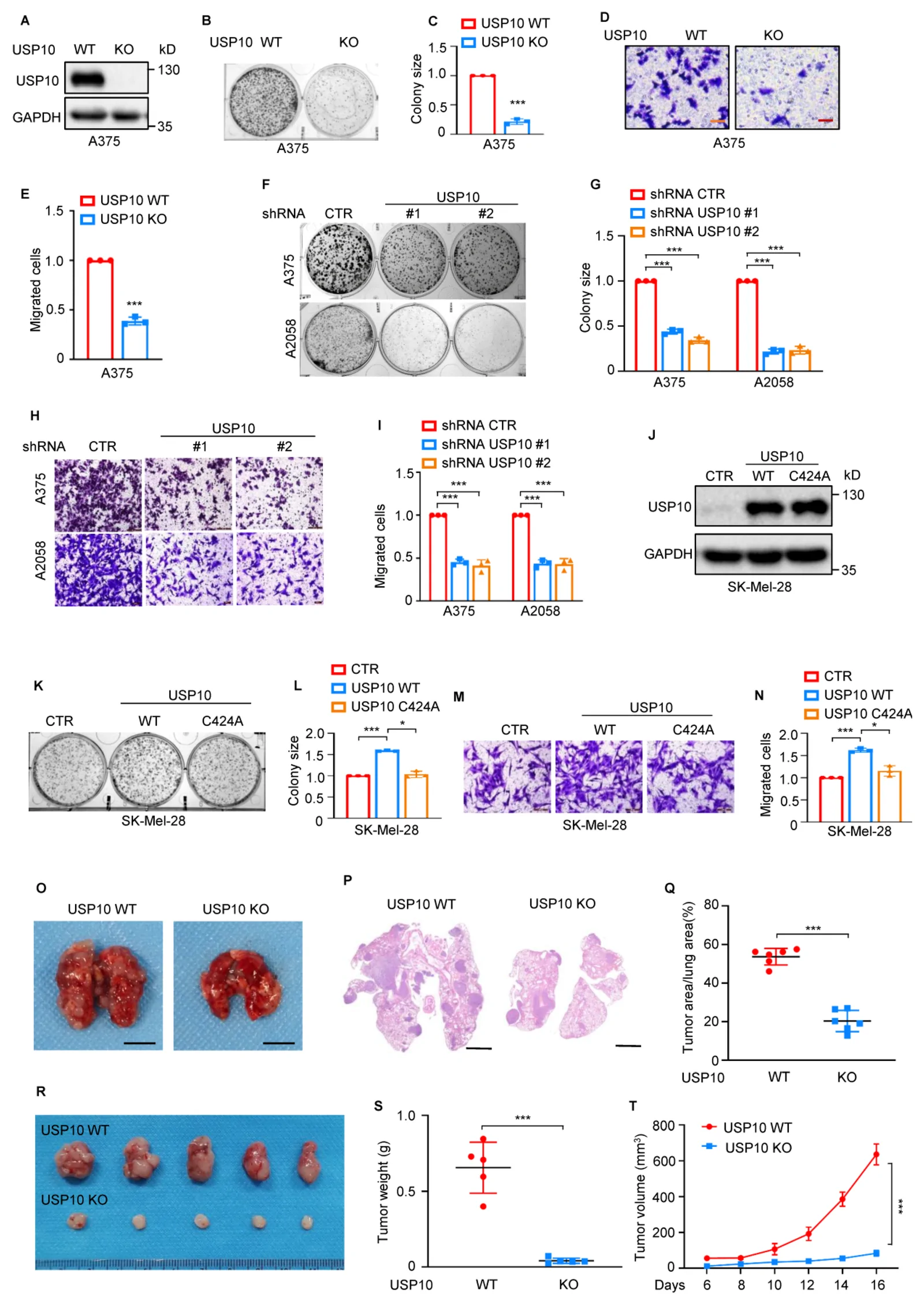
** USP10 accelerates melanoma metastasis and proliferation*.*** (A) Western Blotting was used to detect USP10 protein expression in USP10 wild type (USP10 WT) or USP10 knockout (USP10 KO) A375 cells. (B-C) Cell proliferation was assessed using colony formation assay. (D-E) Transwell assay was used to analyze cell migration. (F-G) Colony formation assay was used to detect cell proliferation in A375 and A2058 cells with or without USP10 knockdown. (H-I) Transwell assay was used to assess the cell migration. (J) USP10 WT and CA mutant was overexpressed in SK-Mel-28 cells, and western blotting verified USP10 expression. (K-N) Colony formation and Transwell assay was used to measure cell growth and migration. (O-Q) USP10 WT or USP10 KO A375 cells were respectively injected into the tail vein of nude mice (*n*=6 per group). Representative images of lung tissue (O), HE staining images (P), and assessment of metastatic nodules in each group (Q). (R-T) USP10 WT and KO A375 cells were respectively injected subcutaneously into female nude mice (*n*=5 per group), representative images of xenograft tumours (R), calculation and analysis of tumour weights (S) and volumes (T). The B, D, F, H and K results are representative of three independent experiments, the data are shown as mean ± SD; The Q, S and T data are shown as mean ± SEM. These data analyzed by Student's t-test, *p < 0.05, **p < 0.01, and ***p < 0.001.

**Figure 3:**
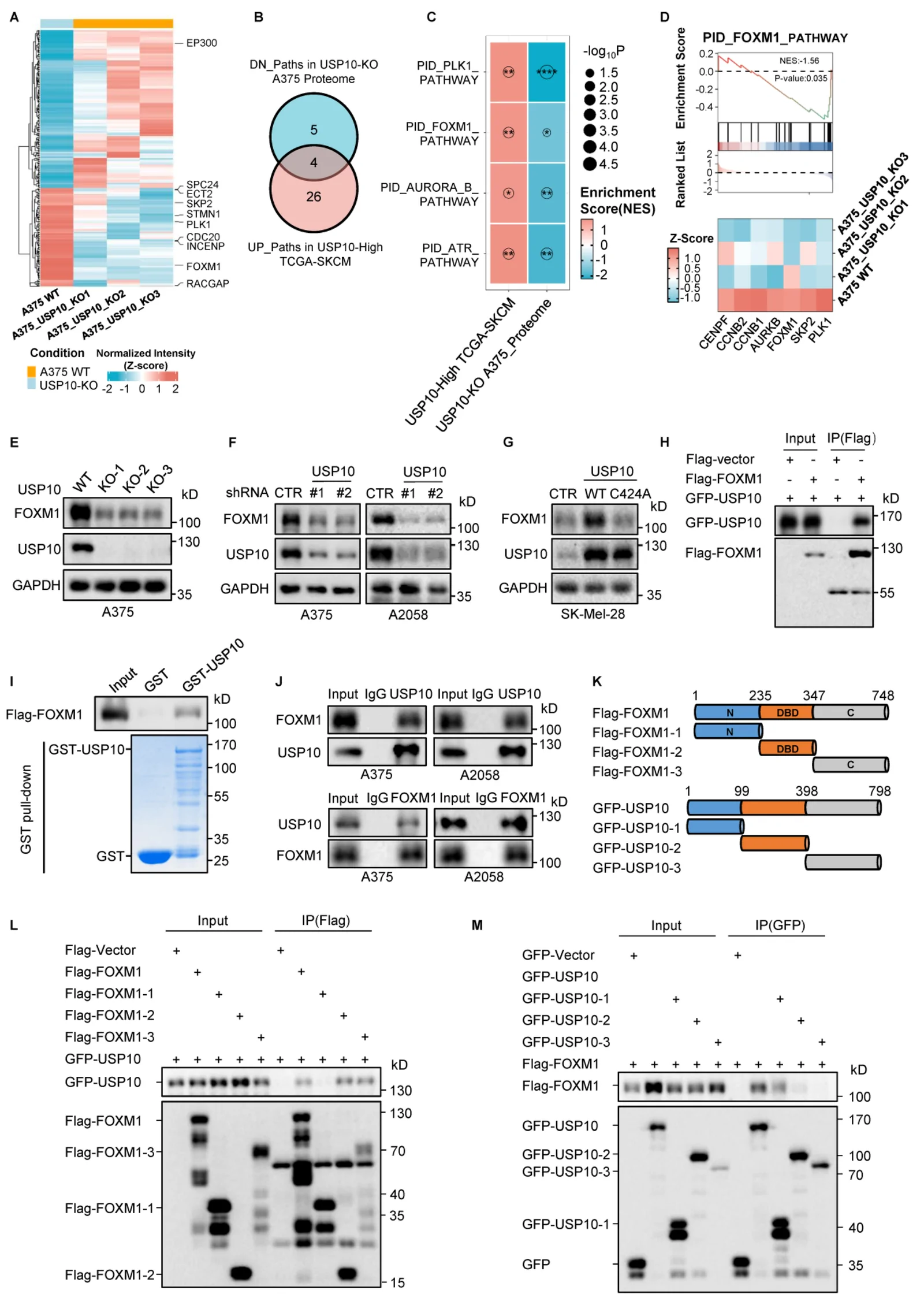
** USP10 interacts with FOXM1 and enhances its expression.** (A) Heatmap visualization of differentially expressed proteins between USP10-KO A375 and A375-WT cells. The color bar denotes Z-score transformed normalized intensity value. PLK1, FOXM1, AURORA_B and ATR pathway related genes were highlighted and labeled. (B-C) Venn diagram showing overlapping pathways identified by Gene Set Enrichment Analysis (GSEA) on USP10-KO A375 versus A375-WT proteomics data and USP10 high versus low TCGA-SKCM RNA-Seq data. Dotplot showing enrichment statistics calculated by GSEA. The color bar denotes normalized enrichment score (NES). Circle size represents the enrichment significance -log10(P). P value< 0.05 are marked with asterisks. *** < 0.001, **<0.01, * < 0.05. (D) GSEA plot of PID_FOXM1_PATHWAY on USP10-KO A375 versus A375-WT proteomics data. Heatmap showing z-score transformed normalized intensity of top genes ranked by log2 (Fold Change) in the pathway. (E) Protein levels of FOXM1 were detected by western blotting in USP10 WT and KO A375 cells (Three USP10-knockout single-cell clones). (F) Western blotting was used to analyze FOXM1 protein levels in USP10 depleting A375 and A2058 cells. (G) USP10 WT and C424A mutants were overexpressed in SK-Mel-28 cells and FOXM1 protein levels were detected. (H) Flag-FOXM1 and GFP-USP10 were co-transfected into 293T cells, and cell lysates were immunoprecipitated using anti-Flag antibodies. (I) The direct interaction between USP10 and FOXM1 was verified by GST pull-down assay. (J) Cell lysates of A375 and A2058 were subjected to immunoprecipitation with control IgG, anti-USP10 and anti-FOXM1 antibodies, followed by the detection of the immunoprecipitates with the indicated antibodies. (K-M) The schematic structure of USP10 and FOXM1. The indicated constructs were transfected into 293T cells and immunoprecipitated using anti-Flag (L) and anti-GFP (M) antibodies. The molecular weight of different truncation mutants: Flag-FOXM1, 114kD; Flag-FOXM1-1, 39kD; Flag-FOXM1-2, 20kD; Flag-FOXM1-3, 63kD; GFP-USP10, 145kD; GFP-USP10-1, 49kD; GFP-USP10-2, 74kD; GFP-USP10-3, 93kD.

**Figure 4:**
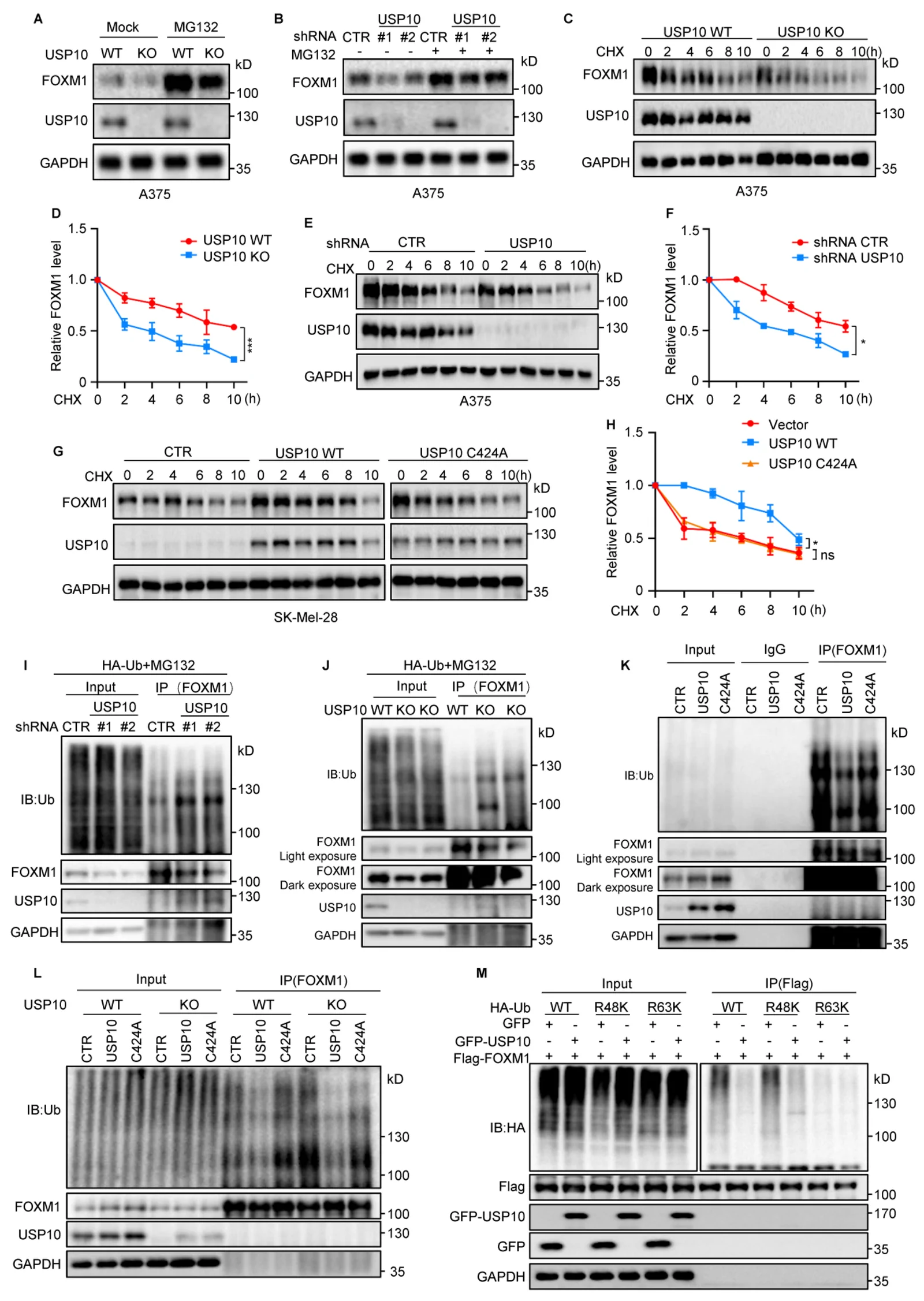
** USP10 maintains FOXM1 stability.** (A) USP10 WT and KO A375 cells were treated with MG132 for 8 h and FOXM1 protein expression levels were detected. (B) A375 cells with or without USP10 knockdown were treated with MG132 and FOXM1 protein levels were detected. (C-F) A375 cells with or without USP10 knockout or knockdown were treated with (50μg/ml) CHX for the indicated times. The protein levels of FOXM1 were determined. (G-H) SK-Mel-28 cells with or without USP10 WT or USP10 C424A overepxression were treated with (50μg/ml) CHX for the indicated times. The protein levels of FOXM1 were determined. (I-J) HA-ub was transfected into A375 cells with or without USP10 knockdown (I) or knockout (J), and 24 h later these cells were treated with MG132 for 8 h. Whole cell lysates were immunoprecipitated using an anti-FOXM1 antibody, and ubiquitination of FOXM1 was detected. (K) HA-ub was transfected into SK-Mel-28 cells overexpressing USP10 WT or C424A mutants, and these cells were treated with MG132 for 8 h. Whole cell lysates were immunoprecipitated using an anti-FOXM1 antibody, and ubiquitinated FOXM1 was detected. (L) The indicated constructs were transfected into USP10 WT or KO A375 cells. FOXM1 ubiquitination were analyzed. (M) HA-Ub (WT or R48K, R63K), Flag-FOXM1 and GFP-USP10 or GFP vector were cotransfected into HEK293T cells. Cell lysates were immunoprecipitated using anti-Flag antibody and ubiquitination of FOXM1 were analyzed. The D, F and H results were representative of three independent experiments, the data were shown as mean ± SD. Student's t-test, *p < 0.05, **p < 0.01, and ***p < 0.001.

**Figure 5:**
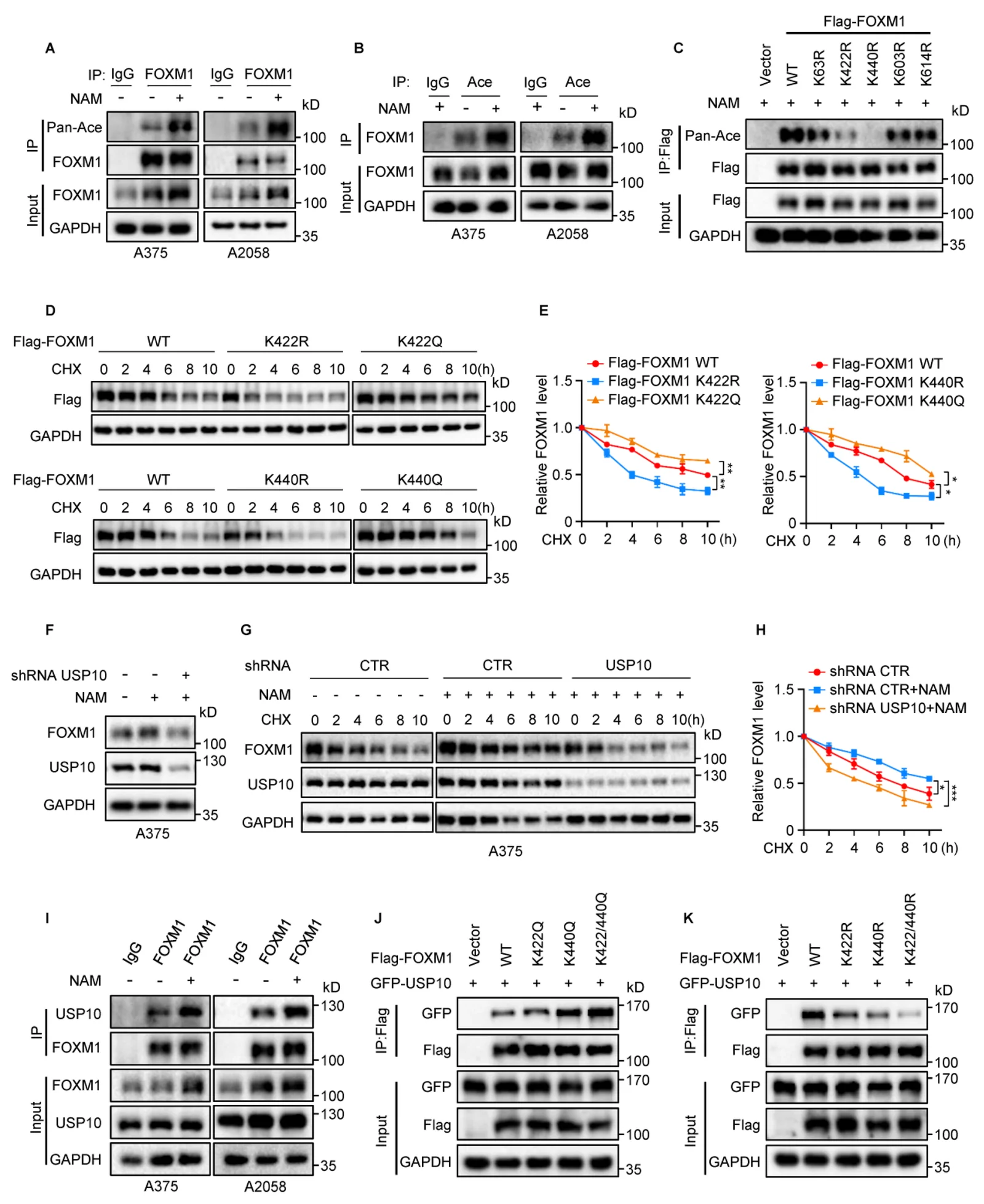
** FOXM1 acetylation enhances interaction with USP10, resulting in FOXM1 stabilization.** (A) Endogenous FOXM1 acetylation was assessed by immunoblotting in A375 and A2058 cells. IgG served as a negative control, and NAM (5 mM, 6 h) was applied to enhance protein acetylation. (B) Acetylated FOXM1 from A375 and A2058 cells was enriched via immunoprecipitation using anti-Pan Ace antibodies, with NAM (5 mM, 6 h) treatment to elevate acetylation levels. (C) The acetylation status of various Flag-tagged FOXM1 mutants was examined in HEK293T cells. Cells were treated with NAM (5 mM, 6 h) to boost acetylation, and acetylated FOXM1 was pulled down using anti-Flag antibodies. (D-E) HEK293T cells expressing Flag-FOXM1 WT, Flag-FOXM1 K422R, Flag-FOXM1 K422Q, Flag-FOXM1 K440R, or Flag-FOXM1 K440Q were exposed to CHX (50 μg/mL) for varying durations to assess protein stability. (F) A375 cells with or without USP10 knockdown were treated with NAM (5 mM, 6 h), followed by measurement of FOXM1 protein expression. (G-H) Following NAM treatment (5 mM, 6 h), A375 cells with or without USP10 silencing were subjected to CHX (50 μg/mL) for different time points, and FOXM1 levels were monitored over time. (I) A375 and A2058 cells were treated with NAM (5 mM, 6 h), and whole-cell lysates were subjected to immunoprecipitation using control IgG or anti-FOXM1 antibodies, with subsequent immunoblot analysis using specified detection antibodies. (J) HEK293T cells were co-transfected with constructs expressing Flag-FOXM1 WT, Flag-FOXM1 K422Q, or Flag-FOXM1 K440Q together with GFP-USP10. Cell lysates were then immunoprecipitated with anti-Flag antibodies and analyzed accordingly. (K) Co-expression of Flag-FOXM1 WT, Flag-FOXM1 K422R, or Flag-FOXM1 K440R with GFP-USP10 in HEK293T cells was followed by anti-Flag immunoprecipitation to evaluate binding interactions. Data in panels E and H were representative of three independent experiments, shown as mean ± SD and evaluated using Student's t-test; *p < 0.05, **p < 0.01, ***p < 0.001.

**Figure 6:**
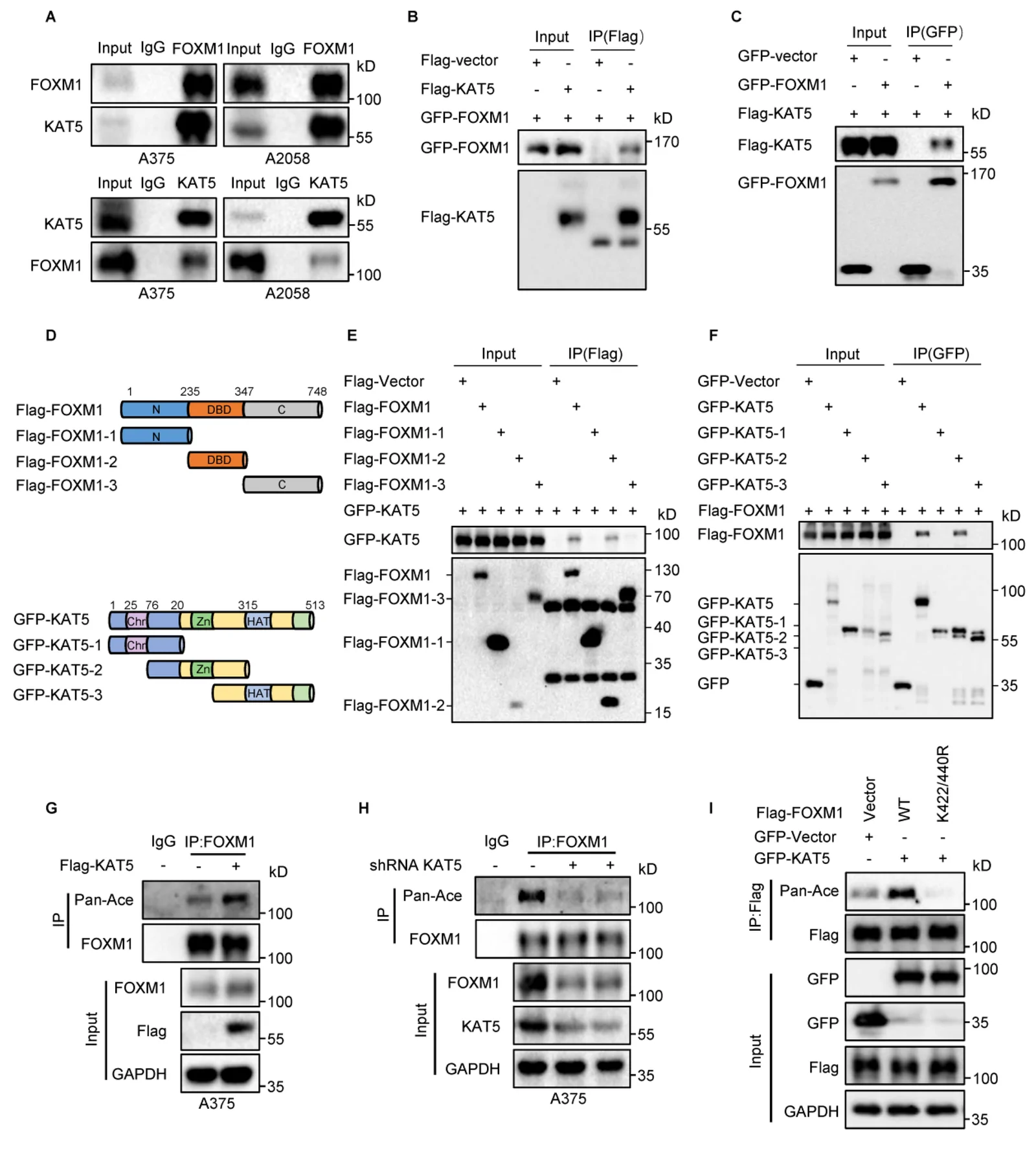
** KAT5 binds to FOXM1 and enhances its acetylation.** (A) Immunoprecipitation was performed on A375 and A2058 cell lysates using control IgG, anti-FOXM1, or anti-KAT5 antibodies, followed by immunoblotting with the specified antibodies to assess protein interactions. (B-C) HEK293T cells were co-transfected with Flag-tagged KAT5 and GFP-tagged FOXM1, followed by immunoprecipitation with anti-Flag or anti-GFP antibodies to confirm their interaction. (D-F) The interaction domains between FOXM1 and KAT5 were determined by transfecting various truncated constructs into HEK293T cells, followed by co-immunoprecipitation using anti-Flag and anti-GFP antibodies. (G) A375 cells were transfected with Flag-KAT5, and acetylated FOXM1 in A2058 cells was Immunoprecipitation with control IgG or anti-FOXM1 antibodies and IB with anti-Pan Ace antibodies. (H) Immunoblotting of FOXM1 acetylation following KAT5 knockdown in A375 cells with IgG as a negative control. (I) Flag-FOXM1 WT or K422/440R mutant were co-transfected into HEK293T cells with or without GFP-KAT5, followed by immunoprecipitation with anti-Flag. Immunoblot analysis used anti-Pan Ace antibodies.

**Figure 7:**
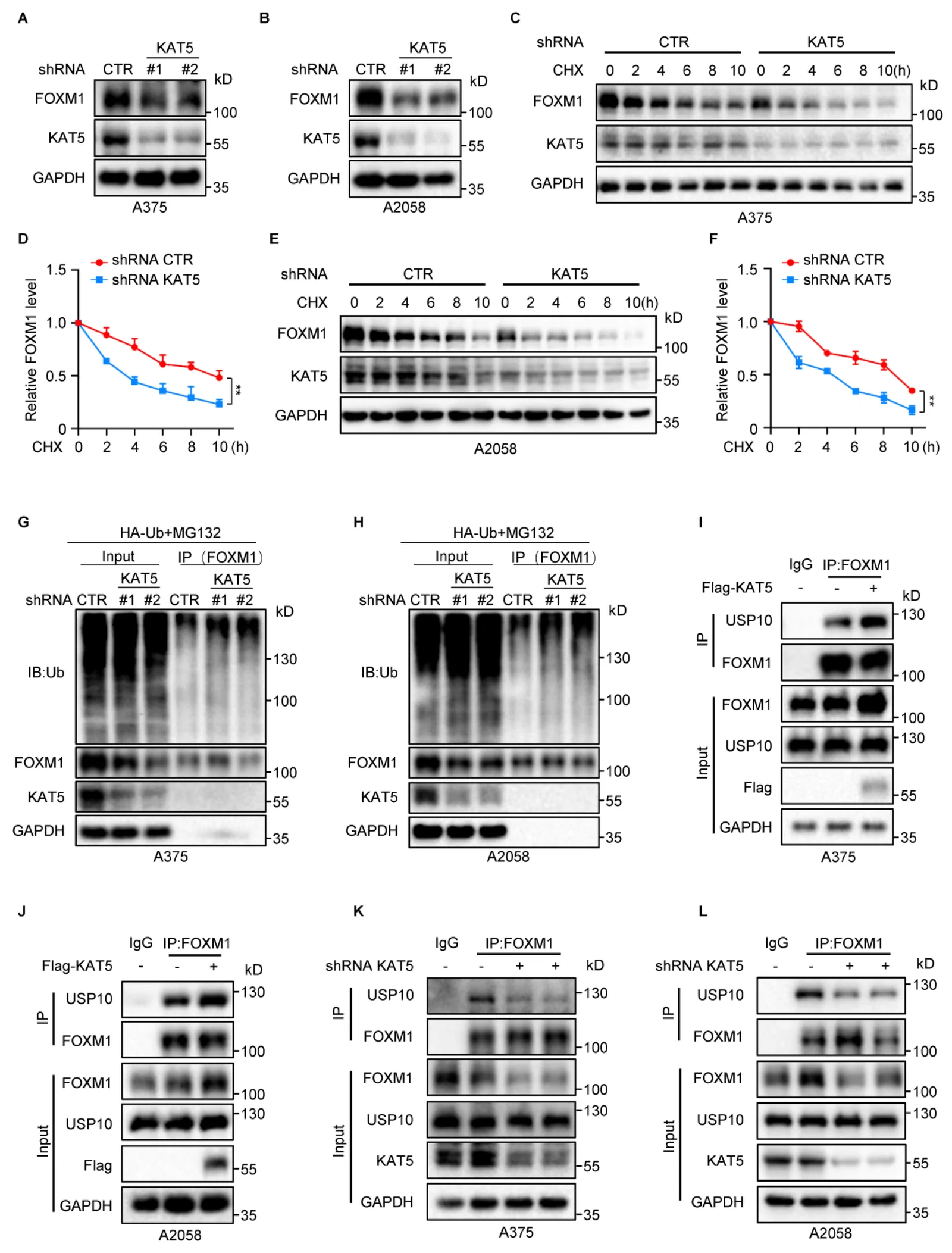
** KAT5 suppresses FOXM1 degradation and enhances its binding to USP10 through regulating FOXM1 acetylation.** (A-B) Western blotting analysis was conducted to evaluate FOXM1 protein expression in A375 and A2058 cells following KAT5 depletion. (C-F) A375 and A2058 cells with or without KAT5 knockdown were treated with cycloheximide (CHX, 50 μg/mL) for varying durations, and FOXM1 protein levels were monitored over time. (G-H) A375 and A2058 cells transfected with HA-Ub and subjected to KAT5 knockdown were treated with MG132 (8 h) 24 h post-transfection. Endogenous FOXM1 was immunoprecipitated, and its ubiquitination status was assessed. (I-J) A375 and A2058 cells transfected with Flag-KAT5 were subjected to immunoprecipitation using control IgG or anti-FOXM1 antibodies, and the resulting complexes were analyzed with FOXM1 and USP10 antibodies. (K-L) Co-immunoprecipitation assays in A375 cells with or without KAT5 silencing were carried out using control IgG or anti-FOXM1 antibodies, followed by immunoblotting for FOXM1 and USP10. The D and F results are representative of three independent experiments, the data are shown as mean ± SD. Statistical analysis of the data in panel D and F were performed using Student's t-test; *p < 0.05, **p < 0.01, ***p < 0.001.

**Figure 8:**
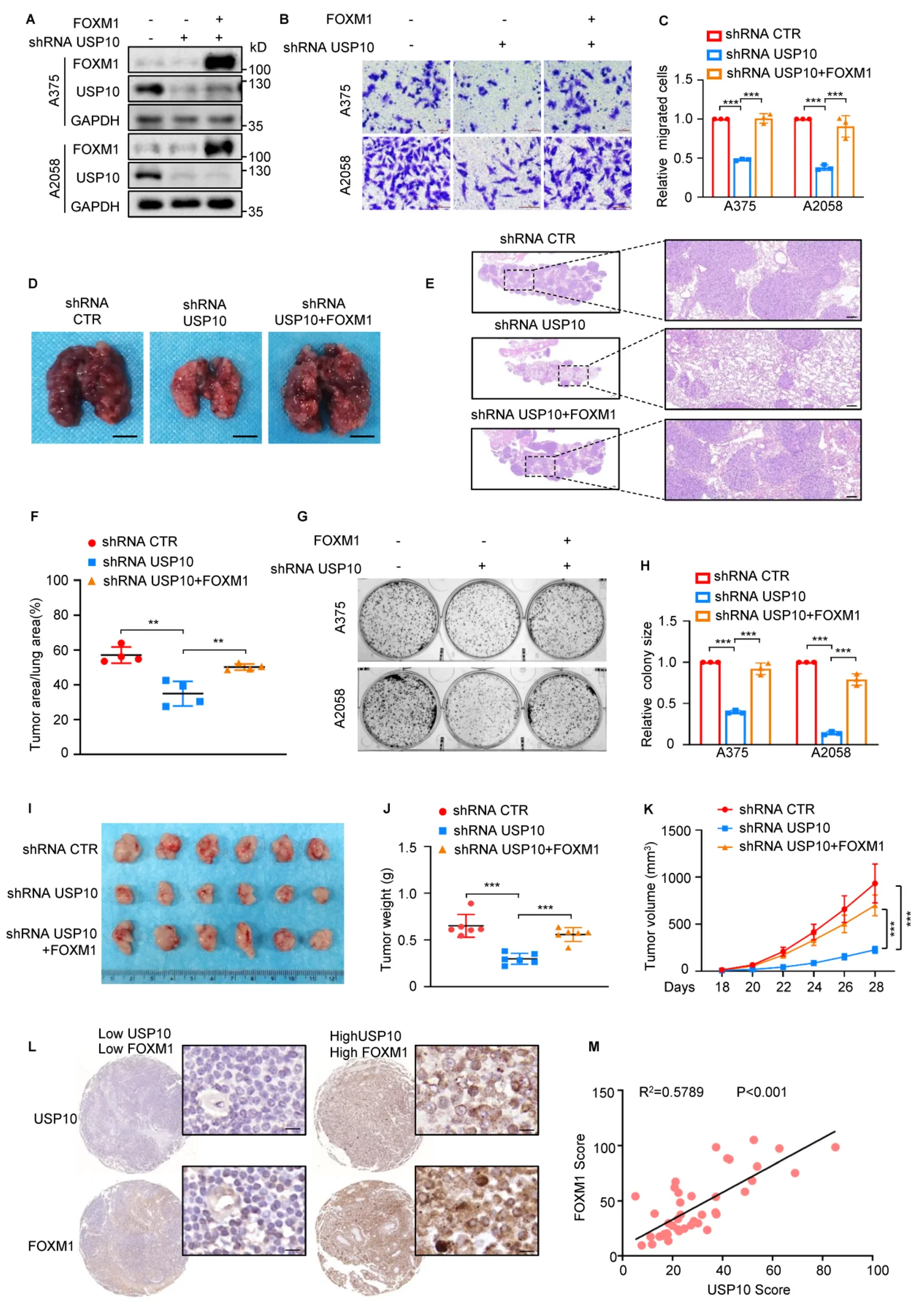
** USP10 facilitates melanoma progression by upregulating FOXM1.** (A) FOXM1 was overexpressed in A375 and A2058 cells with or without USP10 knockdown. The protein levels of USP10 and FOXM1 were detected. (B-C) Transwell assay was used to evaluate cell migration. (D-F) The indicated A375 cells were injected into the tail vein of female nude mice (*n*=4 per group). Representative images of lung tissue (D), HE staining images (E), and assessment of metastatic nodules in each group (F). (G-H) Colony formation assay was used to assess cell proliferation. (I-K) The indicated A375 cells were injected subcutaneously into female nude mice (*n*=6 per group). Representative images of xenograft tumours (I). The calculation and analysis of tumor volume (J) and weight (K). (L) Immunohistochemical staining of melanoma tissue using anti-USP10 and anti-FOXM1 antibodies, selected representative micrographs. Scale bars: 20 μm. (M) The relation between USP10 and FOXM1 expression. The C and H results are representative of three independent experiments. The C, F, H, J, K and M data are shown as mean ± SD and were analyzed by Student's t-test, *p < 0.05, **p < 0.01, and ***p < 0.001.

**Figure 9:**
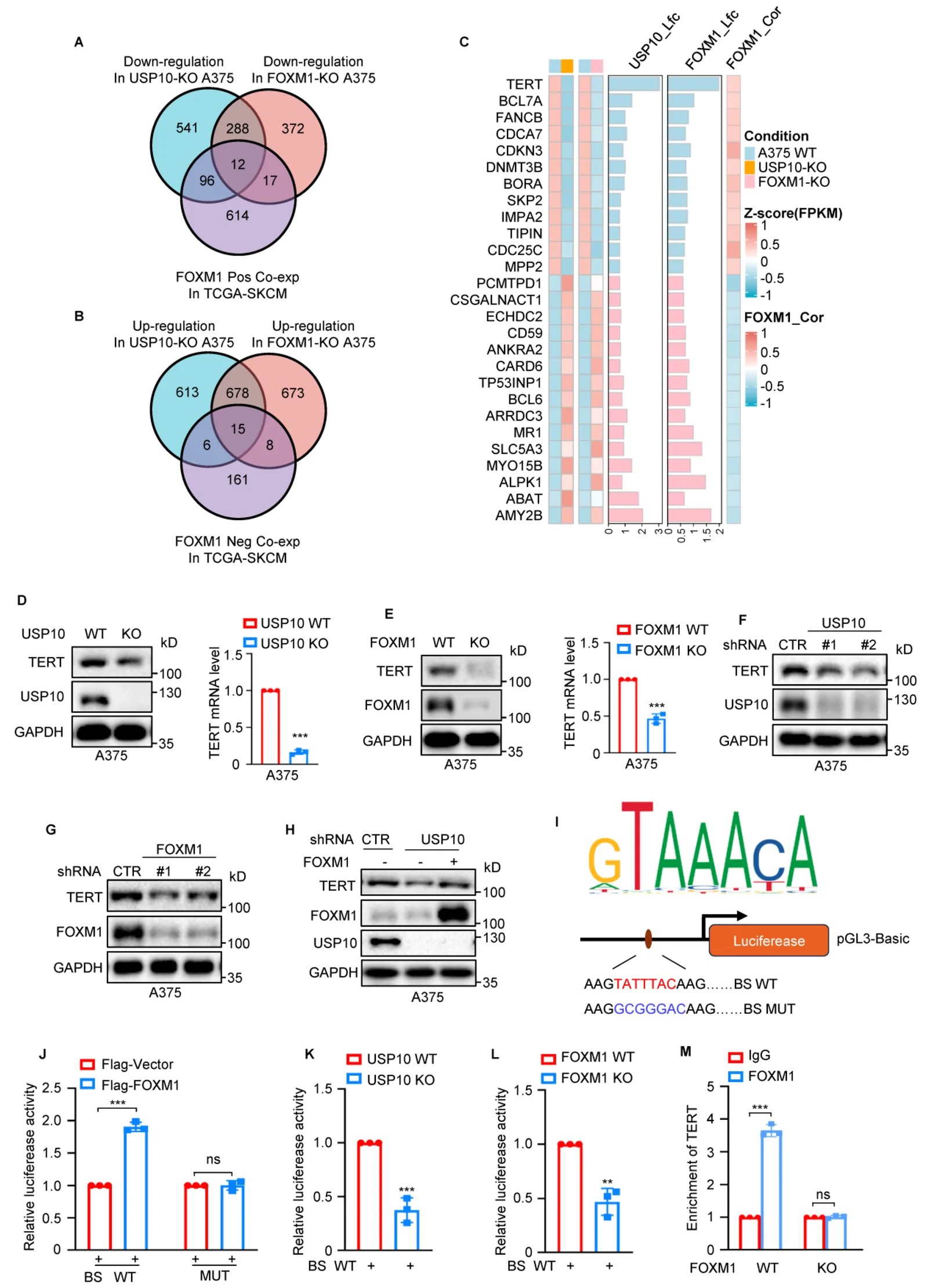
** FOXM1 transcriptional upregulated TERT expression in melanoma cells.** (A-C) Venn diagrams showing overlapping genes and depicting the USP10/FOXM1 axis downstream target screening criteria: 1) down-regulated in USP10-KO A375 cells, 2) down-regulated in FOXM1-KO A375 cells, and 3) positively co-expressed with FOXM1 in TCGA-SKCM cohort. Vice versa, 1) up-regulated in USP10-KO A375 cells, 2) up-regulated in FOXM1-KO A375 cells, and 3) negatively co-expressed with FOXM1 in TCGA-SKCM cohort. Heatmap visualization of overlapping genes. The color bar denotes Z-score normalized FPKM expression value. Barplot showing Log2 (Fold Change) of differential expressed genes and another color bar represents the spearman correlation coefficient of FXOM1-coexpressed genes (absolute r >= 0.3). (D-E) Detection of TERT protein and mRNA using qRT-PCR and western blotting in A375 cells with or without USP10 or FOXM1 knockout. (F-G) TERT protein levels were detected in USP10 or FOXM1 knockdown A375 cells. (H) FOXM1 was overexpressed in USP10 knockdown A375 cells, and then TERT expression was analyzed. (I) Schematic representation of the TERT wild-type binding site (BS WT) and mutant (BS MUT) used in the luciferase assay. (J) The wild-type promoter (BS WT) or the mutant (BS MUT) together with the Renilla luciferase plasmid was individually transfected into 293T cells with or without FOXM1 overexpression. Luciferase activity was measured. (K-L) The BS WT promoter and Renilla luciferase plasmid were transfected into A375 cells with or without USP10 or FOXM1 knockout, followed by measuring the Dual luciferase activity. (M) The FOXM1 binding to the TERT promoter in A375 cells with or without FOXM1 knockout was shown by ChIP analysis. The D, E, J, K, L, and M data are representative of three independent experiments, The results are shown as mean ± SD and were analyzed by Student's t-test, *p < 0.05, **p < 0.01, and ***p < 0.001.

**Figure 10:**
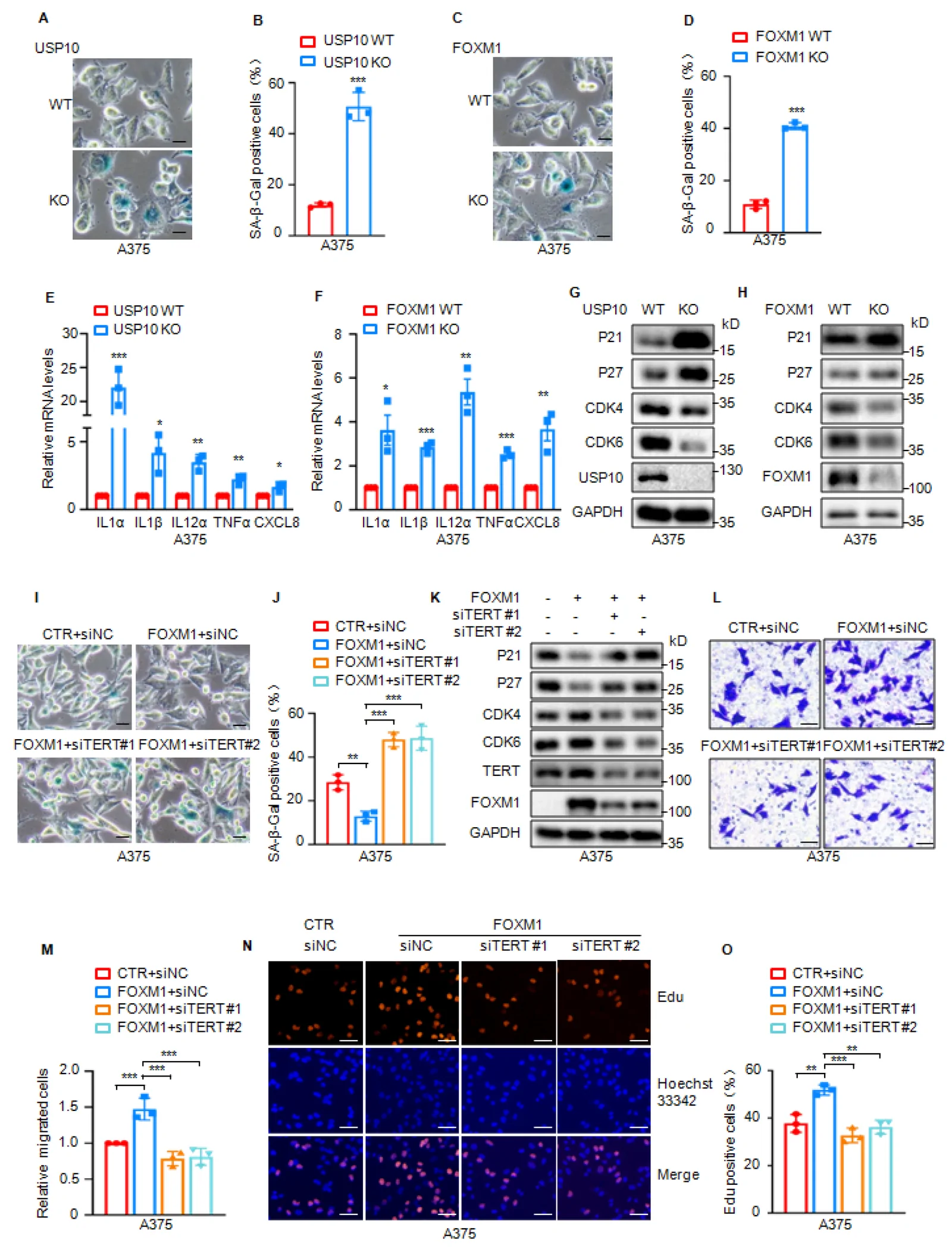
** Deficiency of USP10 and FOXM1 induces melanoma cell senescence through down-regulation of TERT.** (A-D) β-Gal staining was used to measure cell senescence in A375 cells with or without USP10 or FOXM1 knockout. (E-F) The RNA levels of senescence-associated secretory phenotype (SASP) factors were analyzed using qRT-PCR in A375 cells with or without USP10 or FOXM1 knockout. (G-H) The protein levels of P21, P27, CDK4, and CDK6 were analyzed using western blotting in A375 cells with or without USP10 or FOXM1 knockout. (I-J) TERT was knocked down in FOXM1 overexpressing A375 cells and then the cells were treated with doxorubicin (0.25µM /24h) and stained with β-Gal. (K) The protein levels of P21, P27, CDK4, CDK6, FOXM1 and TERT were detected by western blotting. (L-M) TERT was knocked down in FOXM1 overexpressing A375 cells and cell migration was analyzed using Transwell assay (M-N) Edu assay was performed to analyze cell proliferation. The B, D, E, F, J, M, and O data are representative of three independent experiments. The data are shown as mean ± SD and were analyzed by Student's t-test, *p < 0.05, **p < 0.01, and ***p < 0.001.

**Figure 11:**
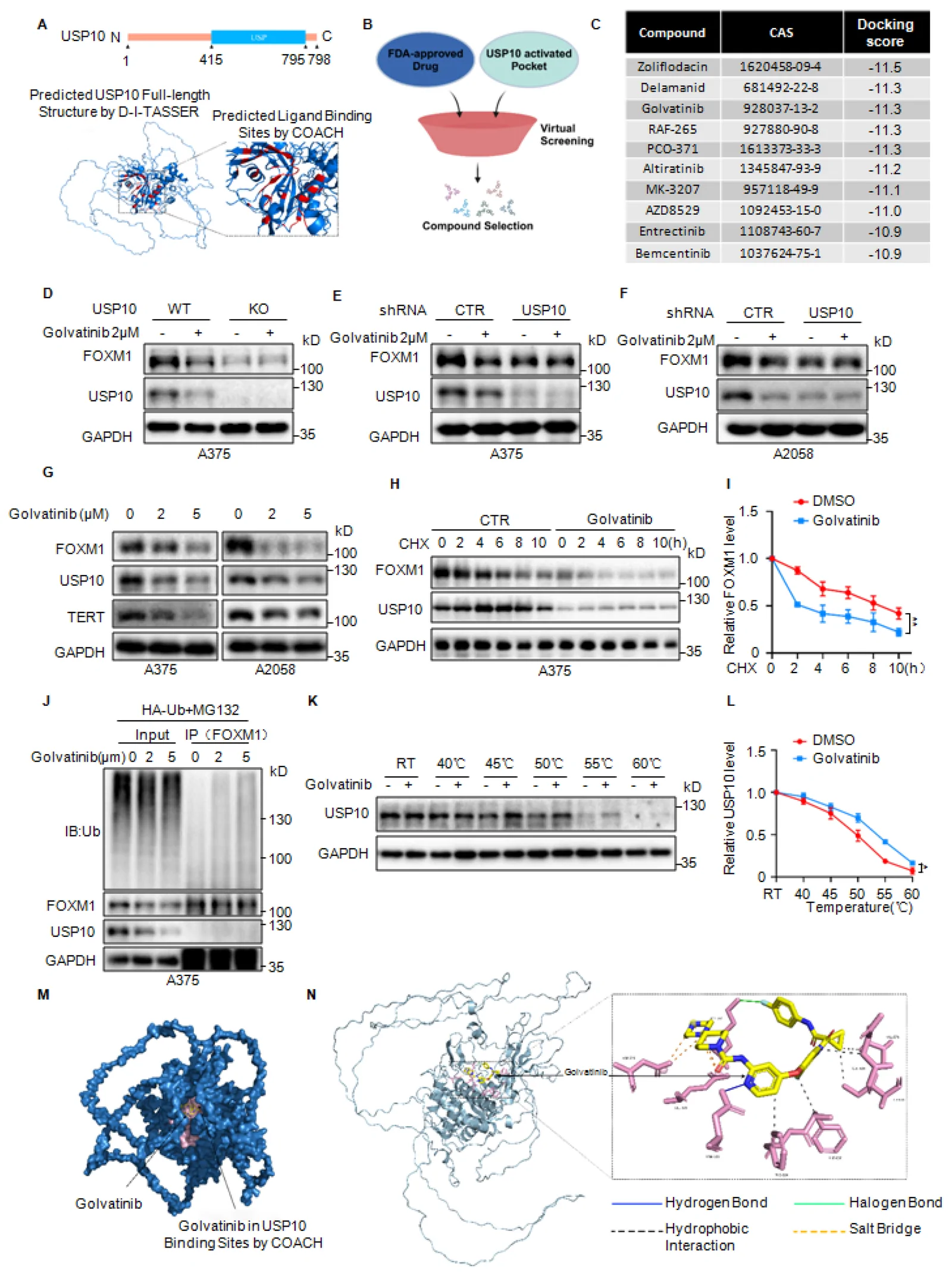
** Identification of Golvatinib binding and inhibition of USP10.** (A) Diagram of human USP10, showing USP domain. Crystal full-length structure of the USP10 was predicted by D-I-TASSER. A blue cartoon representation is shown. Ligand binding sites predicted by COACH are labeled in red. (B-C) Virtual screening of FDA-approved drugs to identify small molecules binding withUSP10 and top 10 hits are listed. (D) Western blotting was employed to detect the expression of FOXM1 and USP10 proteins in USP10 KO and control cells, with and without treatment with 2µM Golvatinib for 24h. (E-F) A375 and A2058 cells with or without USP10 depletion were treated with 2µM Golvatinib for 24h. The expression levels of FOXM1 and USP10 were detected. (G) FOXM1, USP10 and TERT protein levels were detected in A375 and A2058 cells treated with 0, 2 and 5µM Golvatinib. (H-I) A375 cells with or without 2µM Golvatinib treatment for 24h were treated with 20 µg/ml CHX for the indicated times. The half-life of FOXM1 was determined. (J) HA-ub was transfected into A375 cells after the indicated Golvatinib treament, and these cells were then treated with MG132 for 8 h. Whole cell lysates were immunoprecipitated using anti-FOXM1 antibody, and ubiquitination of FOXM1 was detected by western blotting. (K-L) Cellular Thermal Shift Assay showing that Golvatinib attenuated temperature-induced degeneration of the USP10 protein. (M) Surface representation of the structure of the USP10 in complex with Golvatinib. The residues around Golvatinib are highlighted in pink. (N) Overall structure of USP10 in complex with Golvatinib, and Close-up view of the compound binding site highlighting key residues. The I and L data are representative of three independent experiments. The data are shown as mean ± SD and were analyzed by Student's t-test, *p < 0.05, **p < 0.01, and ***p < 0.001.

**Figure 12:**
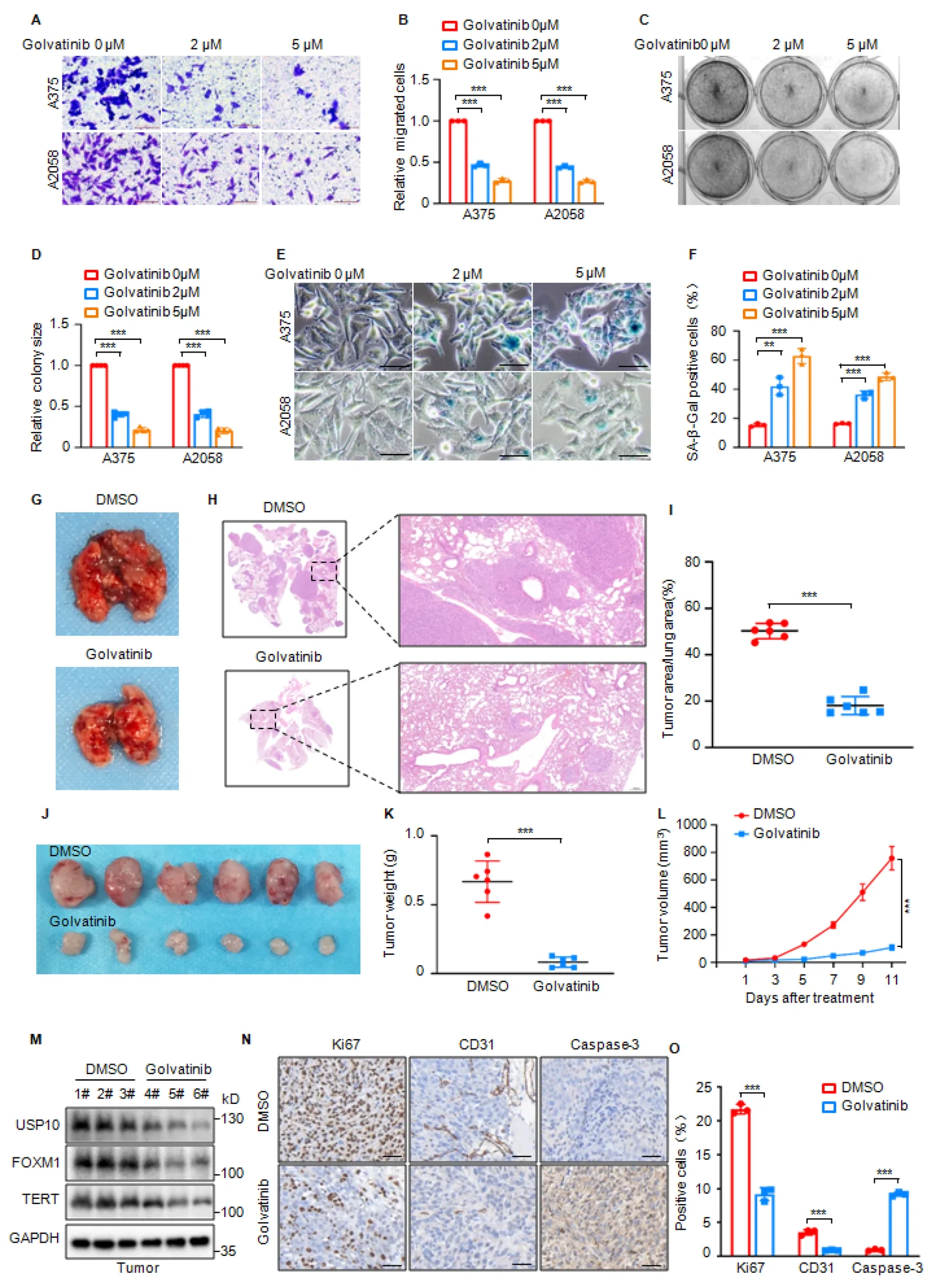
** Golvatinib inhibits melanoma by targeting USP10.** (A-D) Transwell and Colony formation assays were performed to evaluate the migratory and proliferative capacity of the cells treated with Golvatinib. (E-F) β-Gal staining was used to count the proportion of senescence cells after Golvatinib treatment. (G-I) A375 cells was injected into tail vein of female nude mice (*n*=6 per group), The mice were treated with or without Govatinib (50 mg/kg) by gavage once every two days, and the lung tissue was removed three weeks later. Representative images of lung tissue (G), HE staining images (H), and assessment of metastatic nodules in each group (I). (J-L) A375 cells were injected subcutaneously into female nude mice (*n*=6 per group), one week after which gavage administration of Golvatinib (50 mg/kg/day) was initiated and the tumours were removed after 11 days. Representative images of xenograft tumours (J), calculation and analysis of tumour weight (K) and volume (L). (M) Western blotting was used to detect the protein levels of USP10, FOXM1 and TERT in tumour tissue samples. (N-O) IHC analysis was conducted on tumor tissue sections using anti-Ki-67, anti-CD31, and anti-caspase-3 antibodies. Scale bars: 20 μm. The B, D and F data are representative of three independent experiments. The B, D, F, I and O data are shown as mean ± SD and the K and L data are shown as mean ± SEM, all data were analyzed by Student's t-test, *p < 0.05, **p < 0.01, and ***p < 0.001.

## Data Availability

This research used public data from TCGA-SKCM and GTEx. TCGA-SKCM RNA-Seq and clinical data were downloaded from UCSC XENA (https://xenabrowser.net/datapages/). Curated progression-free survival (PFS) data was obtained from an integrated TCGA Pan-Cancer Clinical Data Resource (TCGA-CDR). The data that support the findings of this study are available from the corresponding author upon reasonable request.
