# Supplementary material for: USP10 stabilizes FOXM1 through KAT5-mediated acetylation to suppress cell senescence and promote melanoma malignant progression
Source: Int J Biol Sci. 2026 Sep 10;22(14):8029–52. doi: 10.7150/ijbs.136120 (PMC13618225; doi:10.7150/ijbs.136120)
Supplement: Supplementary file 1 [file ijbsv22p8029s1.pdf]

Supplementary Figure 1

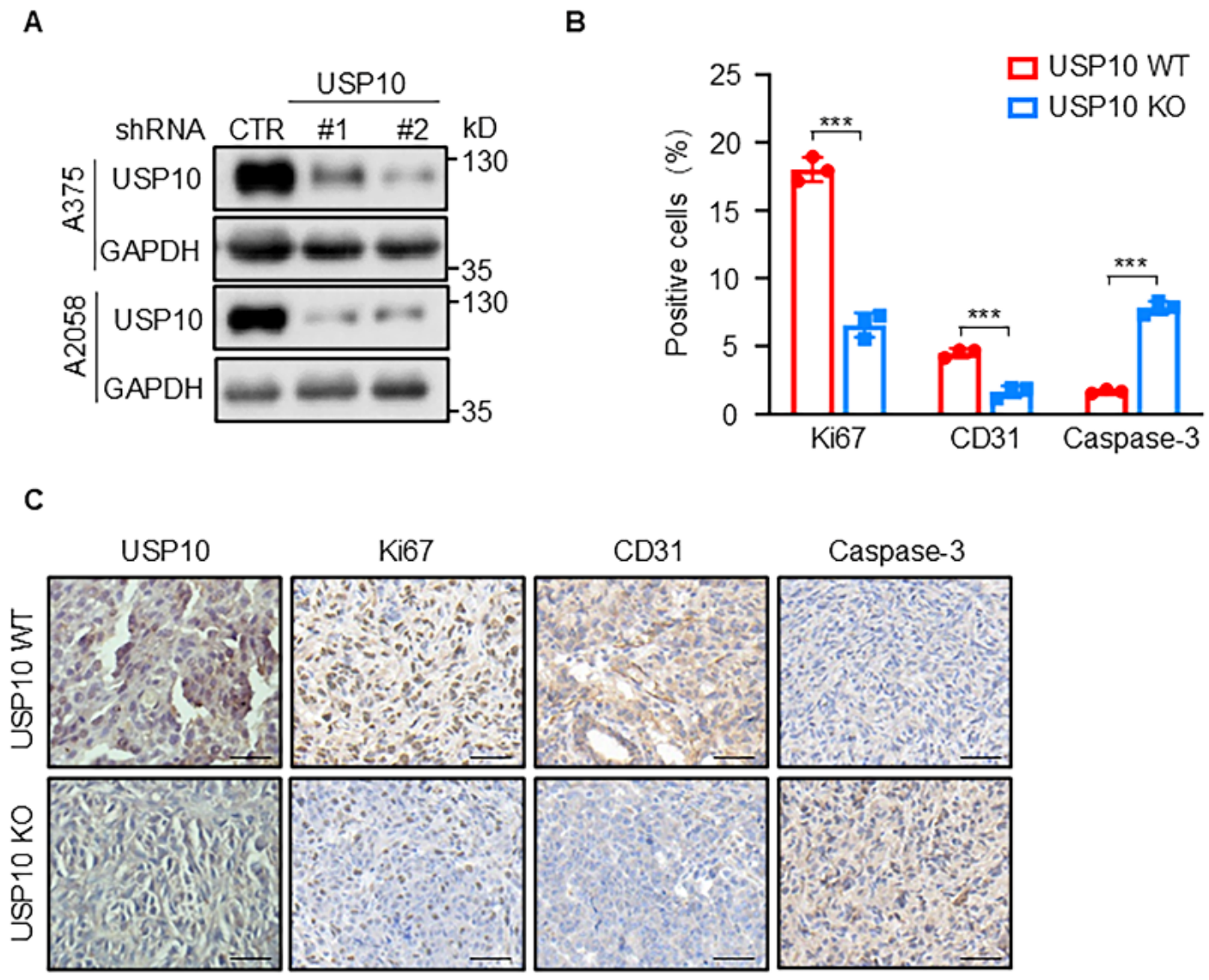

(A) USP10 was knocked down in A375 and A2058 cells and USP10 expression was detected by Western Blotting. (B-C) IHC analysis was conducted on tumor tissue sections using anti-USP10, anti-Ki-67, anti-CD31, and anti-caspase-3 antibodies. Scale bars: 20 μm. The D data is shown as mean ± SD and was analyzed by Student's t-test, *p < 0.05, **p < 0.01, and ***p < 0.001.

Supplementary Figure 2

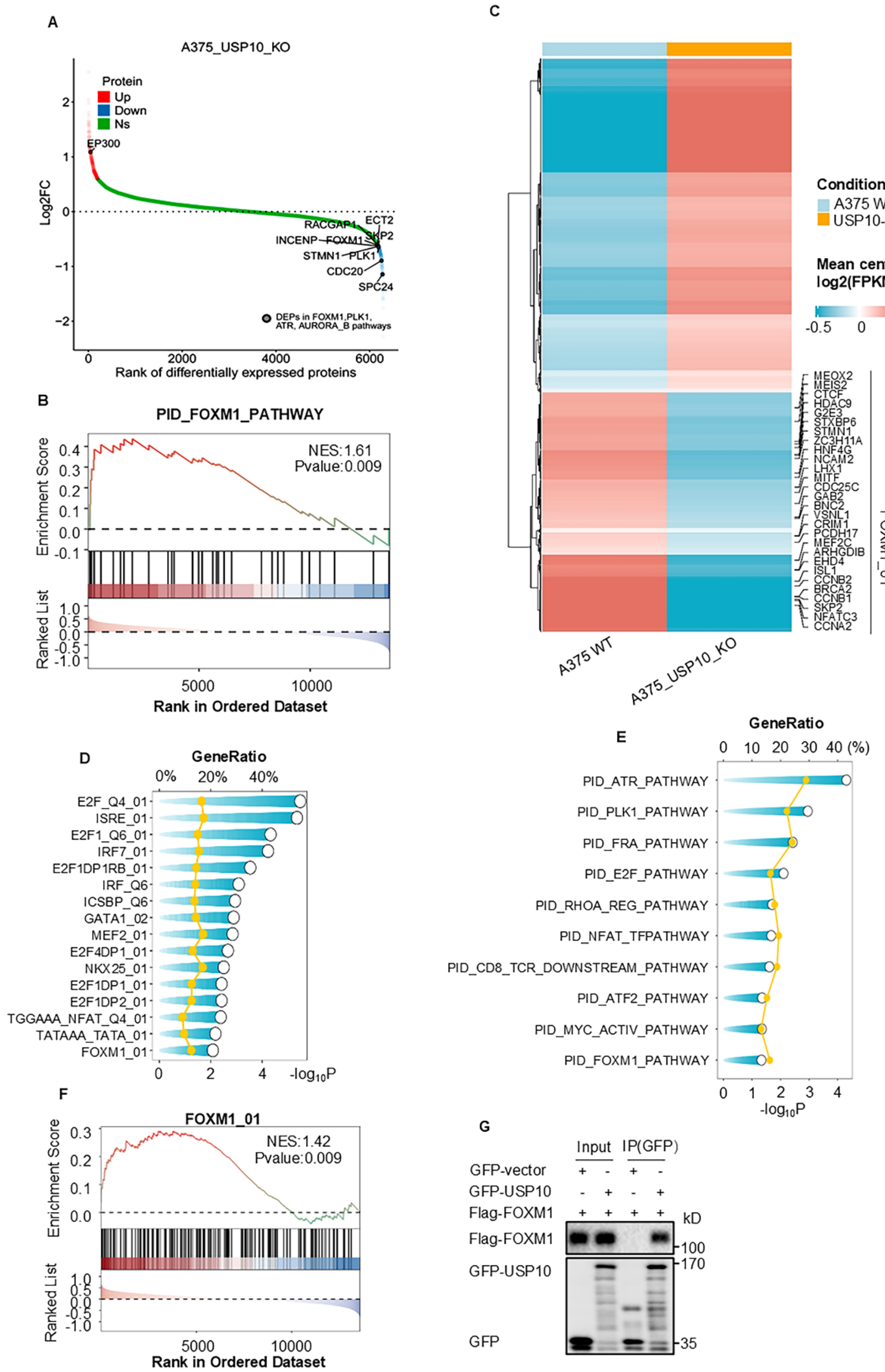

(A) The scatter plot showed the altered proteins in USP10 KO versus WT cells. Altered proteins mapping to PID_FOXM1_PATHWAY, PID_PLK1_PATHWAY, PID_ATR_PATHWAY, PID_AURORA_B_PATHWAY were highlighted and labeled

with gene symbols. (B) Gene set enrichment analysis (GSEA) of PID_FOXM1_PATHWAY in USP10 high versus USP10 low melanoma patients from TCGA-SKCM cohort. (C) Heatmap visualization of differentially expressed genes between USP10-KO A375 and A375-WT cells. The color bar denotes mean centered log2(FPKM+1) expression value. Down-regulated genes mapping to PID_FOXM1_PATHWAY and FOXM1_01 transcription factor targets were marked in orange and purple line and labeled with gene symbols. (D) Transcription factor target enrichment analysis for significantly down-regulated genes between USP10-KO A375 and A375-WT cells. The dual x-axis represents enrichment significance -log10(P value) calculated by hypergeometric test and proportion of significantly down-regulated genes mapping to the corresponding enriched transcription factor respectively. P values < 0.05 was considered statistically significant, and only enriched transcription factors with P values < 0.01 were shown. (E) PID pathway enrichment analysis for significantly down-regulated genes between USP10-KO A375 and A375-WT cells. The dual x-axis represents enrichment significance -log10(P value) calculated by hypergeometric test and proportion of significantly down-regulated genes mapping to the corresponding enriched PID pathway respectively. (F) Gene set enrichment analysis (GSEA) of FOXM1_01 transcription factor targets in USP10 high versus USP10 low melanoma patients from TCGA-SKCM cohort. (G) Flag-FOXM1 and GFP-USP10 were co-transfected into 293T cells, and cell lysates were immunoprecipitated using anti-GFP antibodies.

Supplementary Figure 3

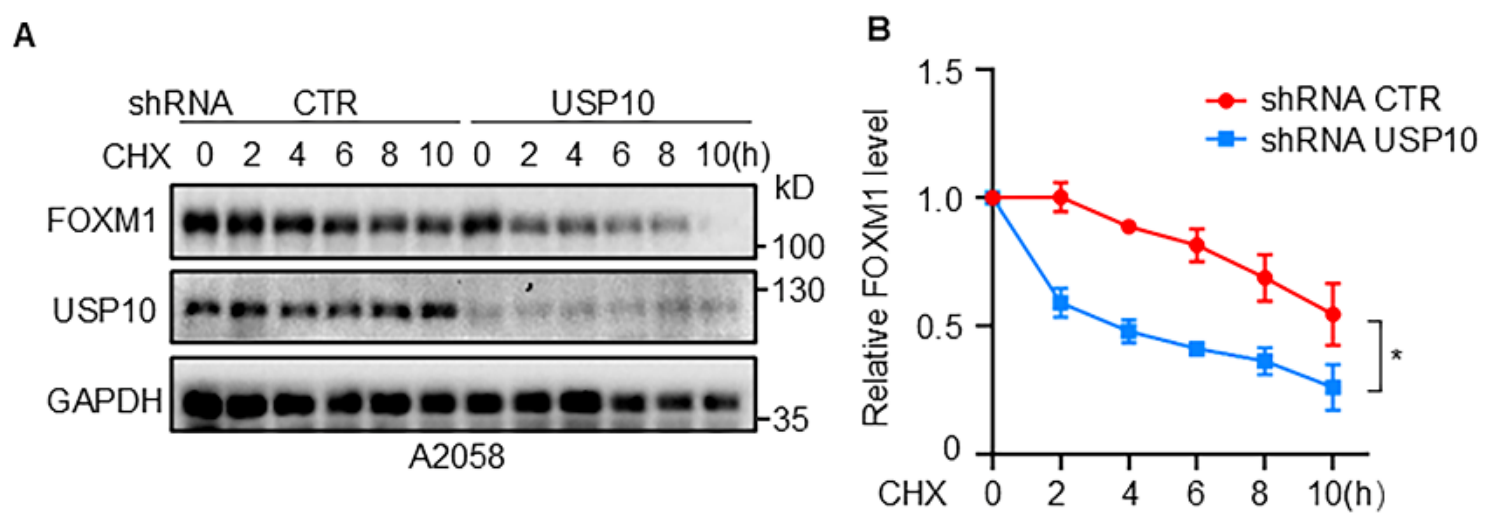

(A-B) A2058 cells with or without USP10 knockdown were treated with 50µg/ml CHX for the indicated times. The half-life of FOXM1 was determined. B data is representative of three independent experiments and data is shown as mean ± SD. Student's t-test, B data were analyzed by Student's t-test, *$p < 0.05$, **$p < 0.01$, and ***$p < 0.001$.

Supplementary Figure 4

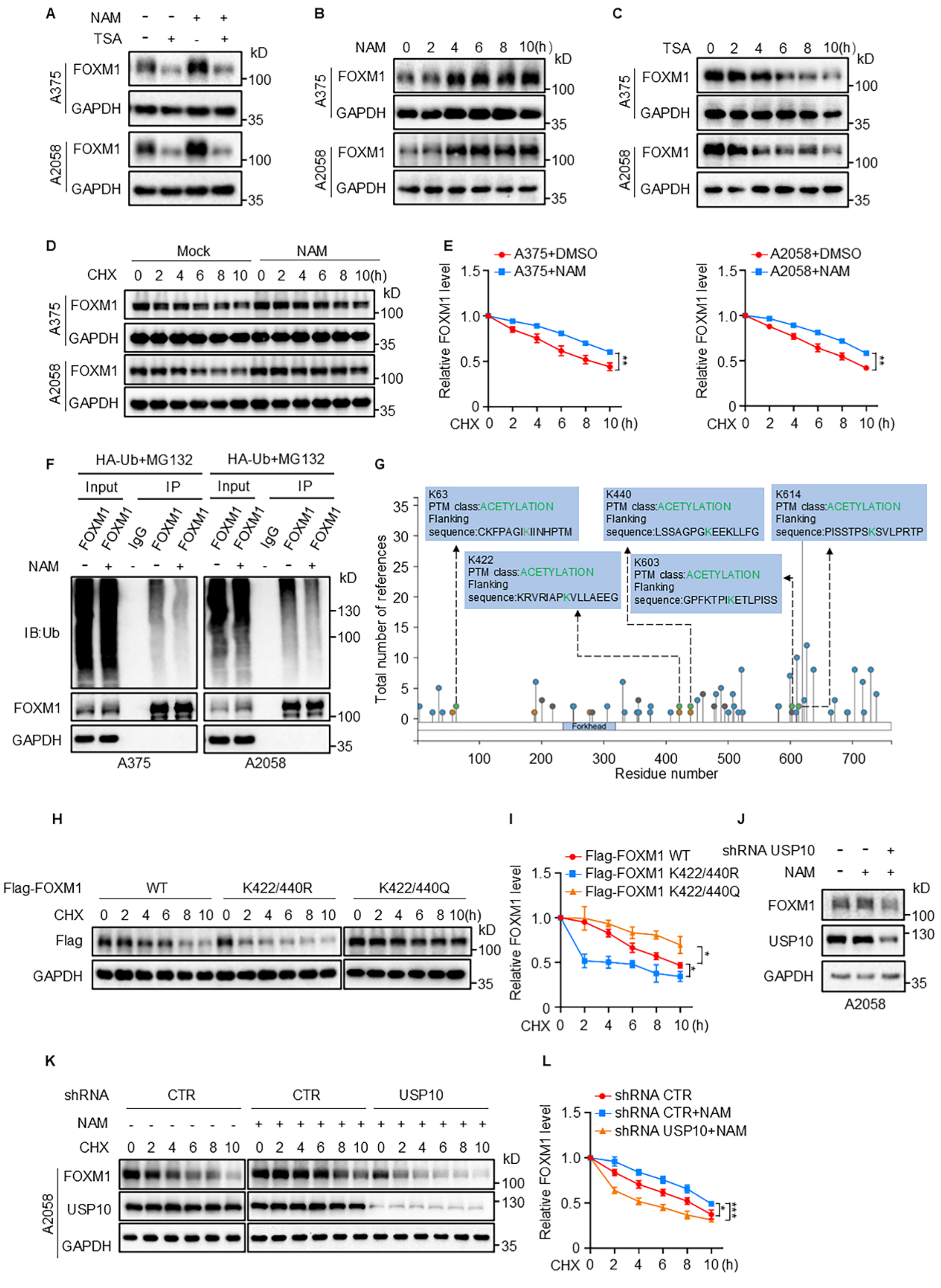

(A) Western blotting was used to detect FOXM1 protein levels after NAM (5mM) and TSA (1μM) treatment for 6 h in A375 and A2058 cells. (B) A375and A2058 cells were treated with NAM (5mM) for the indicated times and FOXM1 protein

expression levels were detected. (C) Western blotting was used to detect FOXM1 protein levels after TSA (1 μM) treatment at different time points. (D-E) After treated with NAM (5mM, 6h), A375 and A2058 cells were treated with (50μg/ml) CHX for the indicated times. (F) HA-Ub was transfected into A375 and A2058 cells, immunoblot of FOXM1 ubiquitination A375 and A2058 cells after MG132(10μM, 8h) and NAM (5mM, 6h) treatment, with IgG as a negative control. (G) Analysis of proteomic databases indicates that 5 lysine residues of FOXM1 are potentially acetylated. (H-I) HEK293T cells expressing Flag-FOXM1 WT, Flag-FOXM1 K422/440R, or Flag-FOXM1 K422/440Q were exposed to CHX (50 μg/mL) for varying durations to assess protein stability. (J) A2058 cells with or without USP10 knockdown were treated with NAM (5mM, 6h) and FOXM1 protein levels were detected. (K-L) After treated with NAM (5mM, 6h), A2058 cells with or without USP10 knockdown were treated with (50μg/ml) CHX for the indicated times. The protein levels of FOXM1 was determined. The E, I and L data are representative of three independent experiments. The data are shown as mean ± SD and were analyzed by Student's t-test, The E, I and L data were analyzed by Student's t-test, *p < 0.05, **p < 0.01, and ***p < 0.001.

Supplementary Figure 5

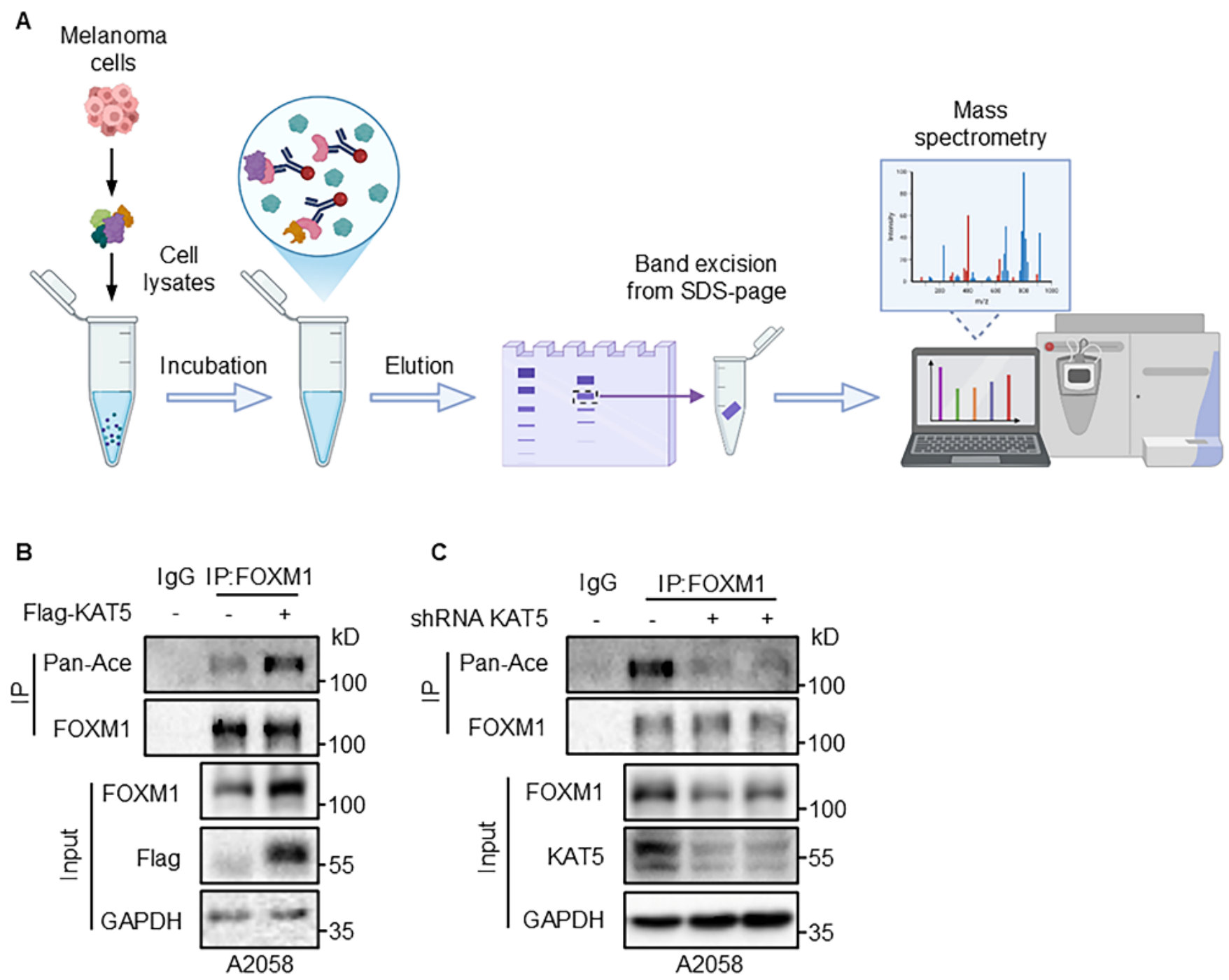

(A) Schematic diagram of the identification of FOXM1 acetyltransferase by mass spectrometry. (B) A2058 cells were transfected with Flag-KAT5, and acetylated FOXM1 in A2058 cells was IP with control IgG or anti-FOXM1 antibodies and IB with anti-Pan Ace antibodies. (C) Immunoblot of FOXM1 acetylation following KAT5 knockdown in A2058 cells with IgG as a negative control.

Supplementary Figure 6

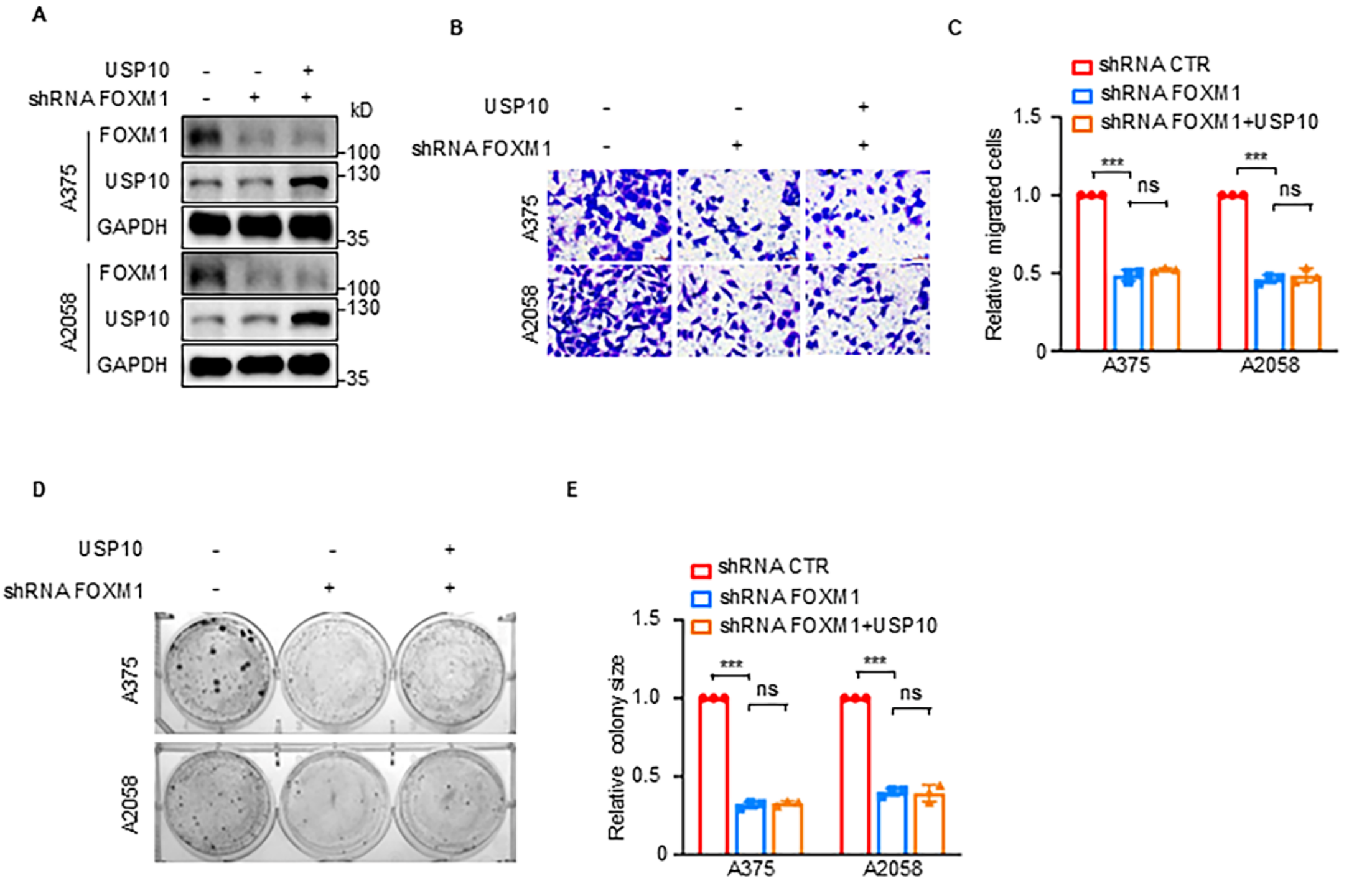

(A) USP10 was overexpressed in A375 and A2058 cells with or without FOXM1 knockdown. The protein levels of USP10 and FOXM1 were detected. (B-C) Transwell assay was used to evaluate cell migration. (D-E) Colony formation assay was used to assess cell proliferation. The D and F data are shown as mean ± SD and was analyzed by Student's t-test, *p < 0.05, **p < 0.01, and ***p < 0.001.

Supplementary Figure 7

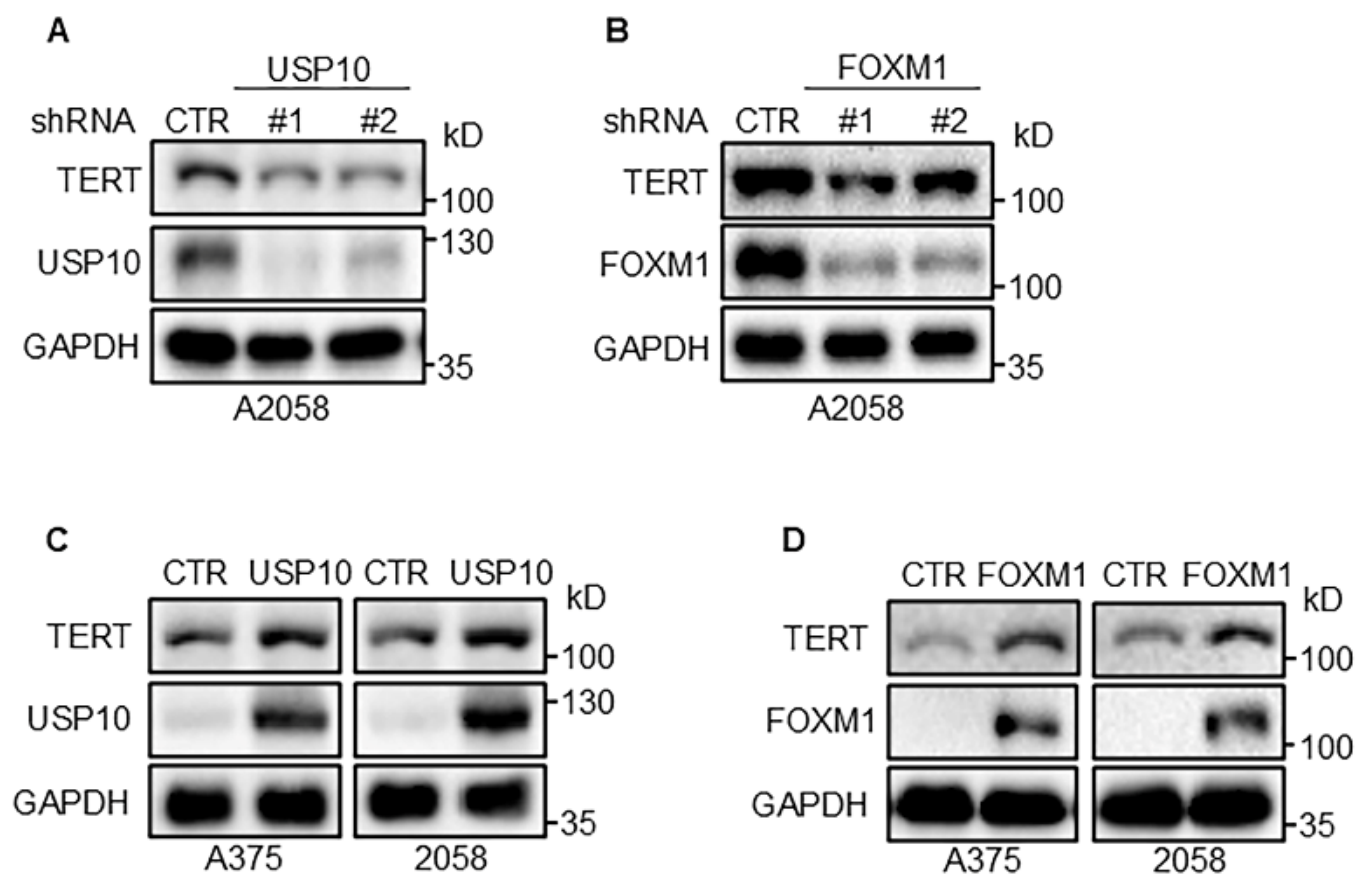

(A-B) TERT protein expression was detected in USP10 or FOXM1 depleting A2058 cells. (C-D) USP10 or FOXM1 was overexpression in A375 and A2058 cells and TERT expression was detected by Western Blotting.

Supplementary Figure 8

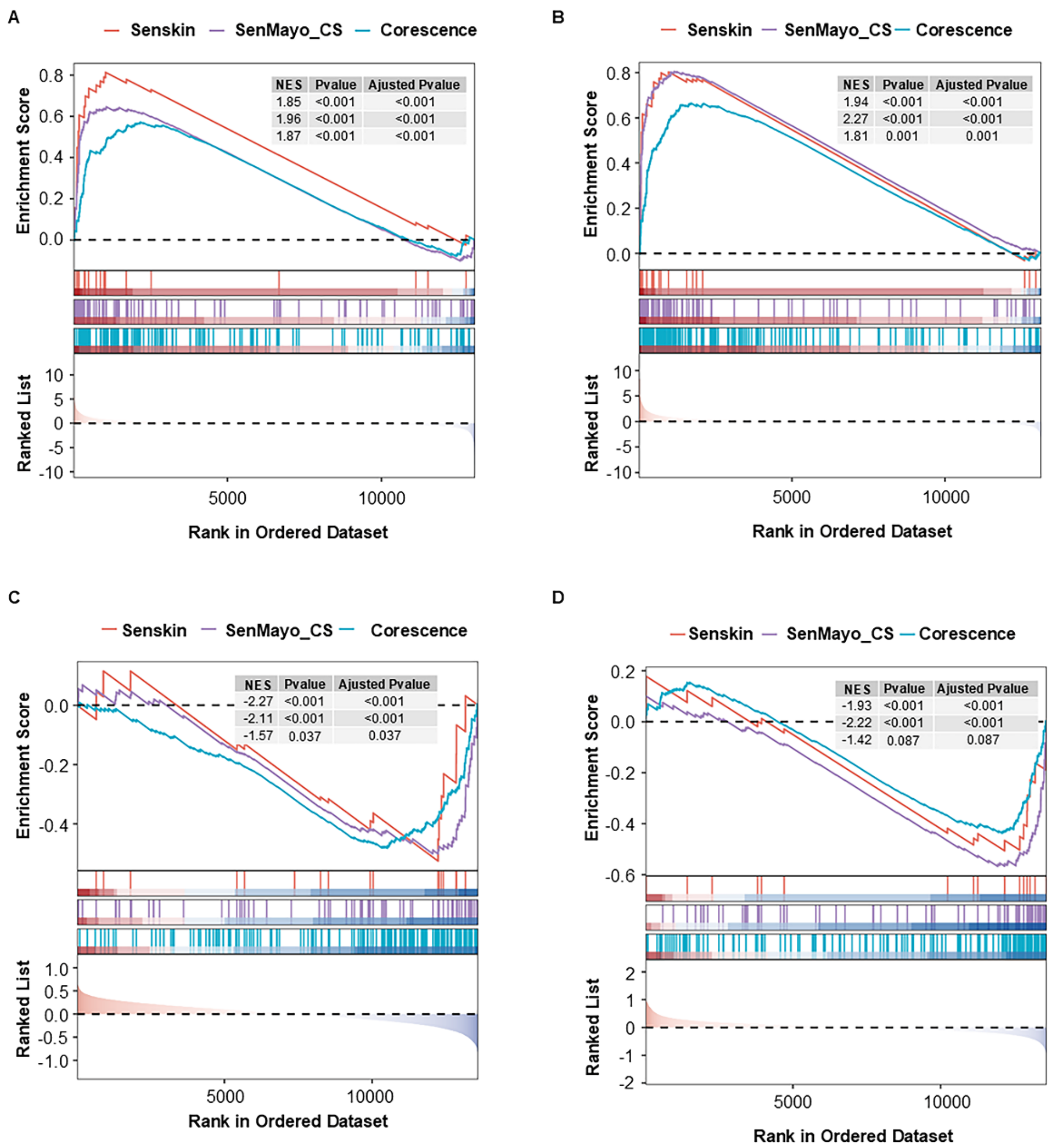

(A-B) Cellular senescence gene set enrichment analysis in USP10- and FOXM1-deficient A375 cells. (C-D) Cellular senescence gene set enrichment analysis in USP10 high versus USP10 low melanoma patients from TCGA-SKCM. Cellular senescence gene set enrichment analysis in FOXM1 high versus FOXM1 low melanoma patients from TCGA-SKCM.

Supplementary Figure 9

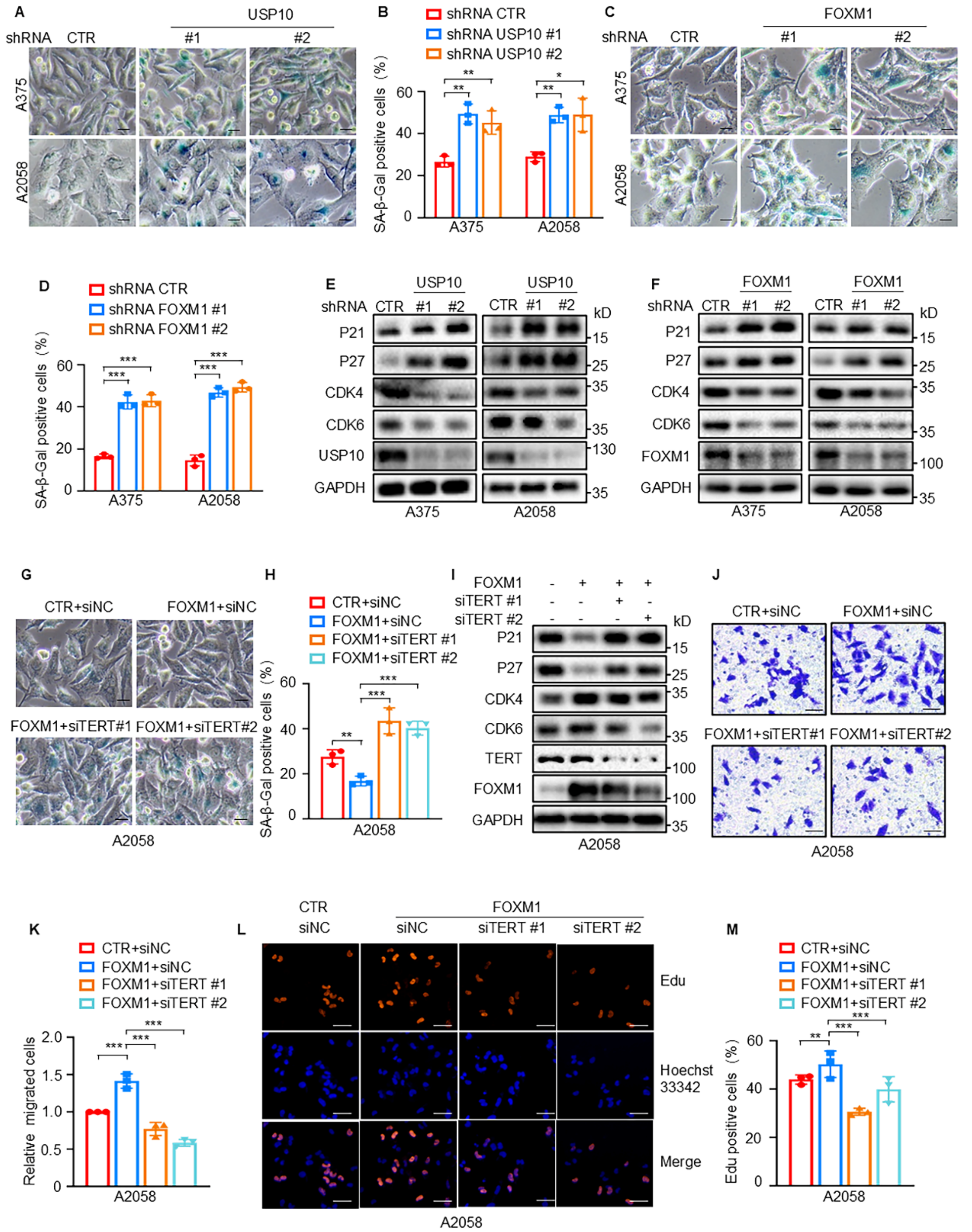

(A-D) β-Gal staining was used to assess cell senescence in A375 and A2058 cells with or without USP10 or FOXM1 knockdown. (E-F) The expression levels of P21, P27, CDK4, CDK6 and USP10 were analyzed in A375 and A2058 cells with or without USP10 or FOXM1 depletion. (G-H) TERT was knocked down in FOXM1 overexpressing A375 cells and then the cells were treated with 0.25μM doxorubicin

for 24h and stained with β-Gal. (I) the protein levels of P21, P27, CDK4, CDK6, FOXM1 and TERT were detected by western blotting. (J-K) TERT was knocked down in FOXM1 overexpressing A2058 cells and cell migration was analyzed using Transwell assay (L-M) Edu assay was performed to analyze cell proliferation. The B, D, H, K and M data are representative of three independent experiments. All data are shown as mean ± SD and were analyzed by Student's t-test, *$p < 0.05$, **$p < 0.01$, and ***$p < 0.001$.

Supplementary Figure 10

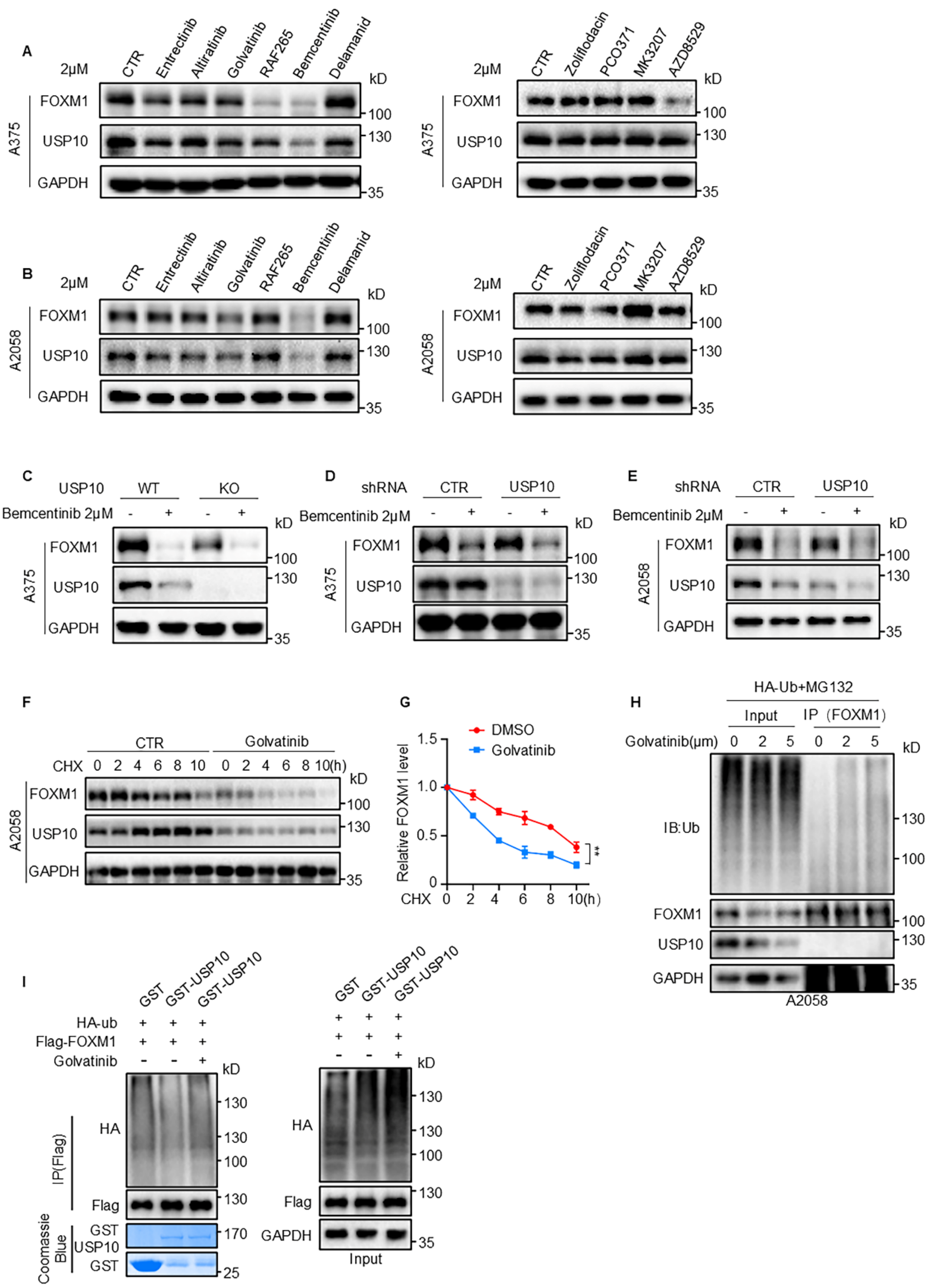

(A-B) FOXM1 and USP10 protein expression was detected by western blotting after treatment of A375 and A2058 cells with different inhibitors (2μM/24h). (C) Western blotting was employed to detect the expression of FOXM1 and USP10 proteins in USP10 WT and KO cells, with and without treatment with 2μM Bemcentinib for 24h.

(D-E) Western blotting was used to detect protein expression of FOXM1 and USP10 in A375 and A2058 cells with or without USP10 knockdown 2μM Bemcentinib for 24h. (F-G) A2058 cells with or without Golvatinib (2μM/24h) treament were treated with 20μg/ml CHX for the indicated times. The half-life of FOXM1 was determined. (H) HA-Ub was transfected into A2058 cells after 2μM Golvatinib treatment for 24h, and these cells were treated with MG132 for 8 h. Whole cell lysates were immunoprecipitated using an anti-FOXM1 antibody, and ubiquitination of FOXM1 was detected by western blotting. (I) Flag-FOXM1 and HA-Ub were co-transfected into 293T cells and purified using a Flag antibody, and GST-USP10 was purified from E. coli BL21 (DE3). The two proteins were then incubated for three hours in deubiquitination buffer at 37 °C, either with or without Golvatinib. The level of ubiquitination of Flag-FOXM1 was detected by Western blotting. The G data are representative of three independent experiments. Data is shown as mean ± SD and was analyzed by Student's t-test, *$p < 0.05$, **$p < 0.01$, and ***$p < 0.001$.

Supplementary Figure 11

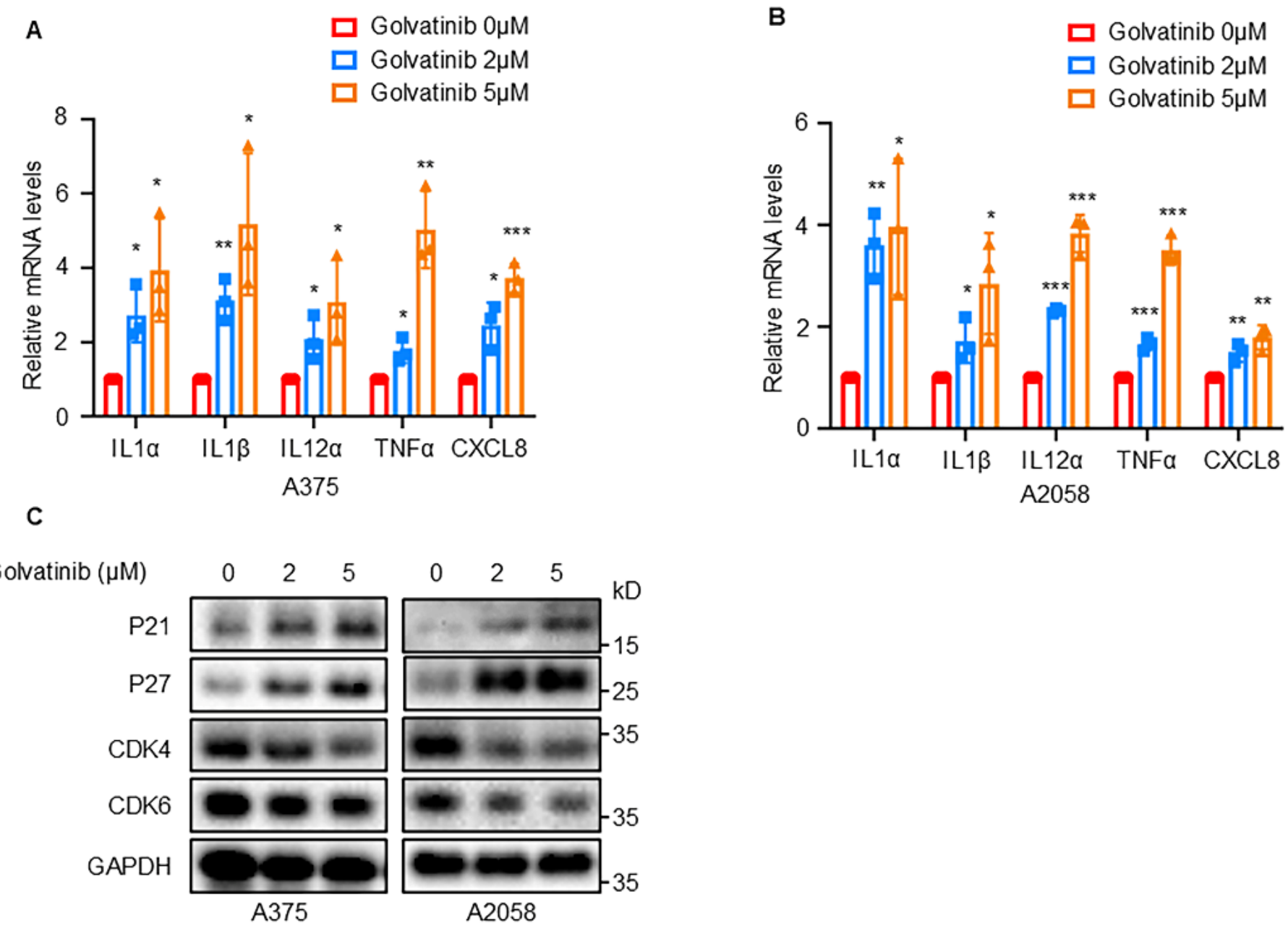

(A-B) A375 and A2058 cells were treated with the indicated Golvatinib and the mRNA levels of SASP factors were assessed by qRT-PCR. (C) Protein levels of P21, P27, CDK4, CDK6 were detected by western blotting. The A and B data are representative of three independent experiments. The A and B data are shown as mean ± SD and were analyzed by Student's t-test, *$p < 0.05$, **$p < 0.01$, and ***$p < 0.001$.

Supplementary Figure 12

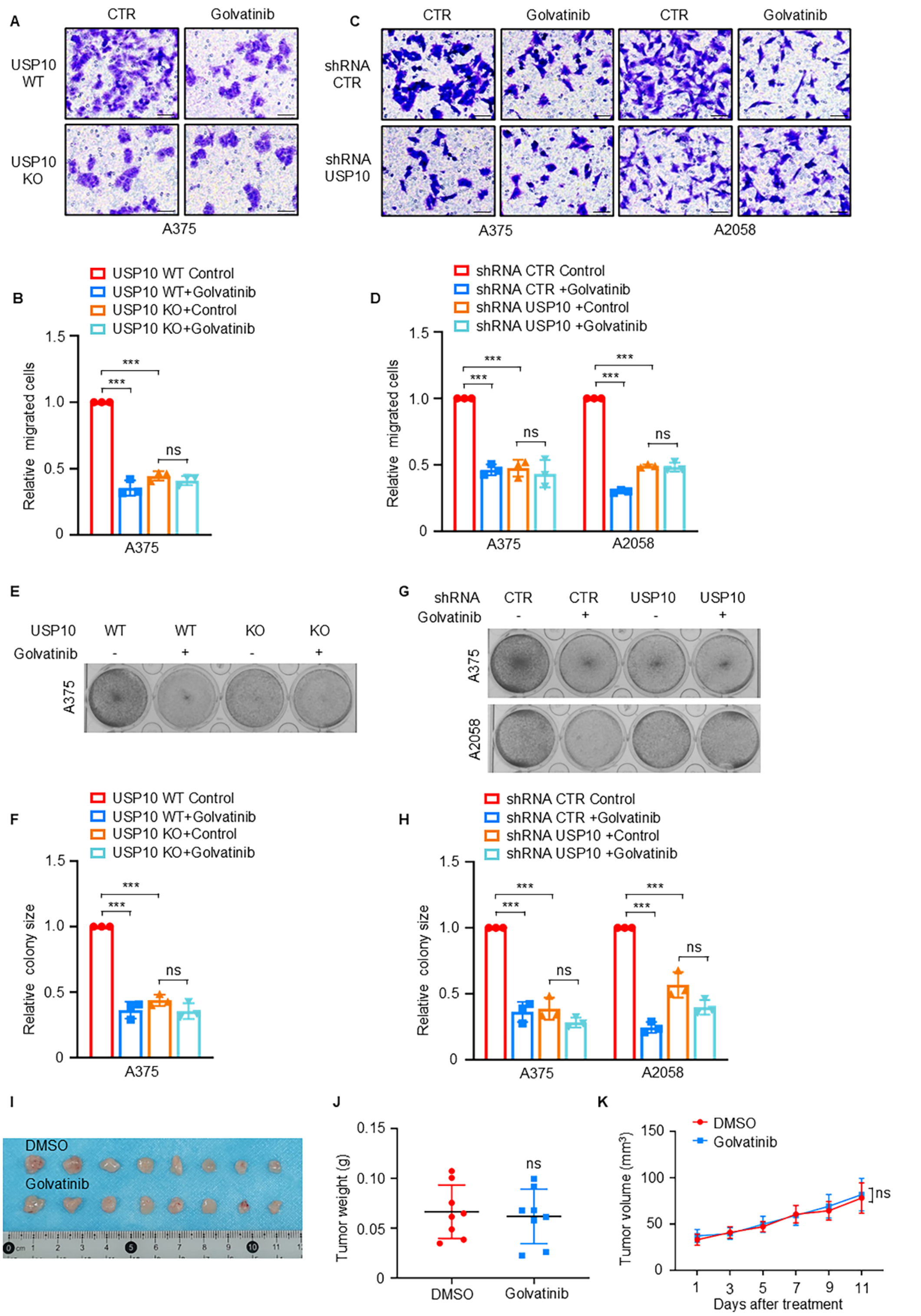

(A-B) USP10 WT and KO A375 cells were treated with 2μM Golvatinib for 24h. Cell migration was analyzed by Transwell assays. (C-D) A375 and A2058 cells with or

without USP10 knockdown were treated with 2μM Golvatinib for 24h. Cell migration was analyzed by Transwell assays. (E-F) USP10 WT and KO A375 cells were treated with 2μM Golvatinib for 24h. Cell proliferation was analyzed by colony formation assays. (G-H) A375 and A2058 cells with or without USP10 knockdown were treated with 2μM Golvatinib for 24h. Cell proliferation was analyzed by colony formation assays. (I-K) USP10 KO A375 cells were injected subcutaneously into female nude mice (*n*=8 per group), one week after which gavage administration of Golvatinib (50 mg/kg/day) was initiated and the tumours were removed after 11 days. Representative images of xenograft tumours (I), calculation and analysis of tumour volume (J) and weight (K). The B, D, F, and H data are representative of three independent experiments. The B, D, F, H, J and K data are shown as mean ± SD and were analyzed by Student's t-test, *p < 0.05, **p < 0.01, and ***p < 0.001.

Supplementary Figure 13

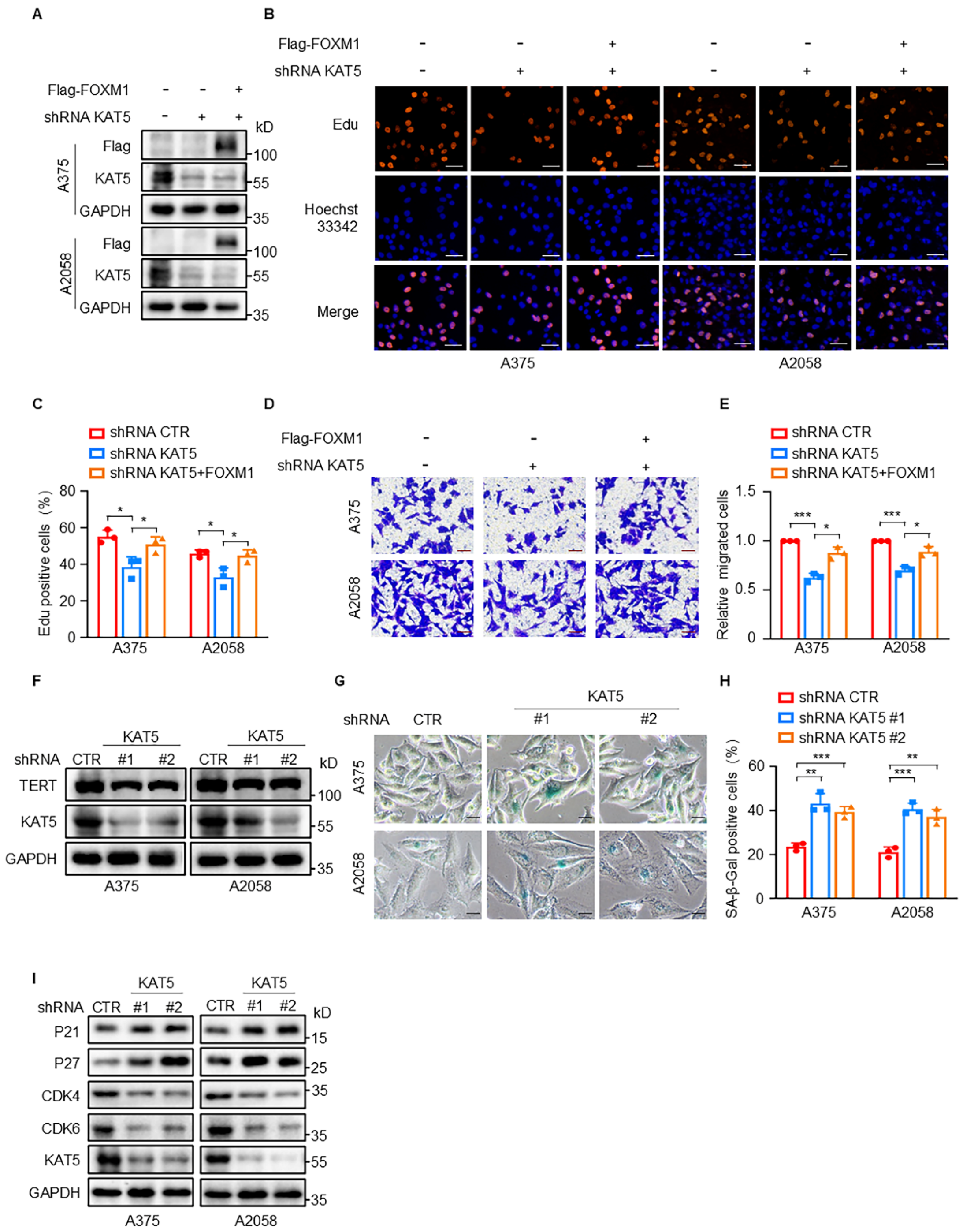

(A) FOXM1 was overexpressed in A375 and A2058 cells with or without KAT5 knockdown. The protein levels of Flag-FOXM1 and KAT5 were detected. (B-C) Edu assay was used to assess the changes of proliferative capacity. (D-E) Transwell assay was used to evaluate cell migration. (F) TERT protein expression was detected in KAT5 depleting A375 and A2058 cells. (G-H) β-Gal staining was used to measure

cell senescence in A375 and A2058 cells with or without KAT5 knockdown. (I) The expression levels of P21, P27, CDK4, CDK6 and KAT5 were analyzed in A375 and A2058 cells with or without KAT5 depletion. The C, E and H data are representative of three independent experiments. The C, E and H data are shown as mean ± SD and were analyzed by Student's t-test, $*p < 0.05$, $**p < 0.01$, and $***p < 0.001$.
