# Supplementary material for: USP10 stabilizes FOXM1 through KAT5-mediated acetylation to suppress cell senescence and promote melanoma malignant progression
Source: Int J Biol Sci. 2026 Sep 10;22(14):8029–52. doi: 10.7150/ijbs.136120 (PMC13618225; doi:10.7150/ijbs.136120)
Supplement: Supplementary file 2 [file ijbsv22p8029s2.pdf]

Supplementary Material and Method

Label-Free Quantitative Proteomics and RNA Sequencing Analysis

For label-free quantitative proteomics, A375 cells with or without USP10 knockout (Three USP10-knockout single-cell clones) were subsequently harvested before being sent to Jingjie PTM Biolab (Hangzhou, China) for further analysis. Briefly, the cells were lysed using a lysis buffer (50 mM Tris-HCl [pH 8.0], 1% Triton X-100, 0.5% Nonidet P-40, 10 mM dithiothreitol, 1% protease inhibitor cocktail, 150 mM NaCl, and 5 mM EDTA) on ice for 30 minutes, followed by centrifugation at 12,000g for 20 minutes at 4°C. The protein solution was precipitated with acetone and reduced with 50 mM dithiothreitol for 1.5 hours at 30°C. Alkylation of the protein solution was performed using 50 mM iodoacetamide for 15 minutes in the dark at room temperature. Subsequently, 100 mM TEAB was added to the urea-containing protein sample, which was then digested overnight with trypsin at a mass ratio of 1:50 (trypsin-to-protein). Finally, the resulting peptides were analyzed via LC–MS/MS using a Thermo Fisher Easy1200-Faims Fusion Orbitrap system.

For database searching, the MS/MS data were processed using the MaxQuant search engine (v.1.6.15.0). Tandem mass spectra were matched against the the human SwissProt database (20422 entries) concatenated with reverse decoy database. Trypsin/P was specified as the cleavage enzyme, allowing up to two missed cleavages. The precursor ion mass tolerance was set to 20 ppm for both the main and first searches, while the fragment ion mass tolerance was set to 0.04 Da. The false discovery rate was adjusted to less than 1%. Normalized intensity was calculated by dividing the mean raw intensity across the sample replicates. Fold change > 1.5 was applied to identify proteins with significant abundance differences upon knockout of USP10 compared to control in A375 cells. Gene set enrichment analysis (GSEA) was performed on pre-ranked gene lists built based on ranking metrics log2FoldChange. Predefined PID pathways (PID subset of CP) were downloaded directly from the Molecular Signatures Database (MSigDB). P values < 0.05 was considered significant. K-nearest neighbor (k-NN) imputation was applied to impute the missing values. The imputation method was implemented in the impute package. Imputed intensity was used for visualization of heatmap.

For RNA sequencing analysis, A375 cells with or without USP10 or FOXM1 knockout were collected and sent to BioMaker for additional processing. RNA extraction, library preparation and sequencing were conducted by BioMaker (Beijing, China). Subsequently, RNA-Seq analysis was performed using in-house BMKCloud platform (www.biocloud.net) developed by BioMaker. Genes with an absolute fold change > 1.5 and a FDR < 0.05 for differential expression comparisons were considered significant. PID pathway and transcription factor target enrichment analyses were conducted by the hypergeometric test using fora function in the fgsea package. Predefined transcription factor targets gene sets (TFT_LEGACY subset of TFT) were also derived from MSigDB. De novo motifs(labeled 'UNKNOWN') that do not map to any known transcription factor were discarded. For redundant enriched transcription factors with different transcription factor bind site(TFBS) motifs, only the most significantly enriched TFBS motif was retained. P values < 0.05 was considered significant.

Bioinformatics analysis

To comprehensively explore the expression pattern and prognostic implications of ubiquitin pathway related gene sets in melanoma, UBQ related genes (validated E1 and E2 enzymes, as well as E3 ligases and their associated adaptor genes) and DUB genes were extracted from previous pan-cancer study on ubiquitin pathway[1]. Predicted UBQ related genes by Hidden Markov model(HMM) were discarded. Very few DUB genes were included in UCH, MJD and MINDY categories, thus they were classified as DUB_others. TCGA-SKCM RNA-Seq (log2(TPM+1)) and clinical data were downloaded from UCSC XENA (https://xenabrowser.net/datapages/). Curated progression-free survival (PFS) data was obtained from an integrated TCGA Pan-Cancer Clinical Data Resource (TCGA-CDR)[2]. We filtered TCGA-SKCM RNA-Seq data to remove low expression genes using zFPKM function in the zFPKM package. We standardized the log scale transcriptome data (log2(TPM+1)) across patients by quantile normalization implemented using normalizeBetweenArrays function in the limma package. Differential expressed genes between metastatic and primary tumor were detected by Non-parametric Mann-Whitney test. Non-parametric Mann-Whitney test was implemented using wilcox.test function in R. The P values were adjusted using the Benjamini & Hochberg (BH) method. BH-adjusted P-value of 0.01 was set

as the threshold for significantly differential expressed genes. For ubiquitin pathway related gene sets enrichment analysis in metastatic versus primary melanoma from TCGA-SKCM cohort, pre-ranked gene lists were built based on modified ranking metrics -log10(P value) * sign(log2FoldChange), since a larger sample size provides more reliable and precise estimates of the population and increases the chances of finding statistically significant results. Based on expression level of USP gene family members, we calculated USP score for each patient in TCGA-SKCM cohort using single sample gene set enrichment (ssGSEA) method implemented in the GSVA package. Patients were divided into two risk groups based on the USP score and optimal cut-off point was determined using surv-cutpoint function in the survminer package. Kaplan-Meier survival curves and log-rank test were used to detect the differences in PFS between USP high/low patients. Multivariate Cox regression analysis was performed to determine independent prognostic genes for PFS after adjusting for TNM stage. 7 patients with stage 0 were removed and stage I was set as reference level for further analysis. Statistical significance among levels for each variable was determined by two-sided Wald test. For Kaplan-Meier survival curves and multivariate Cox regression analysis, patients with less than 1 month of PFS follow-up time were discarded. Gene set enrichment analyses (GSEA) of PID_FOXM1_PATHWAY and FOXM1_01 transcription factor targets in USP10 high versus USP10 low melanoma from TCGA-SKCM cohort were performed on pre-ranked gene lists built based on ranking metrics log2FoldChange. Three cellular senescence gene sets, including SenSkin, SenMayo and CoreScence, were collected from previously studies[3, 4, 5]. For CoreScence gene set, only genes that induce senescence were used according to the original study. Cellular senescence gene set enrichment analyses were performed on pre-ranked gene lists built based on ranking metrics log2FoldChange in USP10- and FOXM1-deficient A375 cells. Cellular senescence gene set enrichment analyses were also performed on pre-ranked gene lists built based on ranking metrics log2FoldChange in USP10(FOXM1) high versus USP10(FOXM1) low melanoma from TCGA-SKCM cohort. All the gene set enrichment analyses (GSEA) mentioned above were implemented using fgsea function in the fgsea package. Melanoma patients from TCGA-SKCM cohort were stratified by USP10(FOXM1) high and low groups using median expression levels. P values < 0.05 was considered significant. Spearman's rank correlation coefficient between FOXM1 expression level and its co-expressed genes was determined in

TCGA-SKCM cohort. Genes with absolute correlation coefficient > 0.3 and BH adjusted P-value < 0.05 were defined as FOXM1 co-expressed genes.

Chromatin immunoprecipitation (ChIP) assay

The A375 cells were crosslinking by exposure to a 1 % form aldehyde for 10 min at room temperature. The ChIP assay was conducted according to the manufacturer's instructions using an anti-FOXM1 antibody and the EZ-ChIP kit (Millipore). The anti-rabbit IgG was employed as a control. The bound DNA frag ments were subjected to eluting and amplification using qRT-PCR.

Quantitative reverse transcription PCR (qRT-PCR) and RT-PCR

Total RNA was extracted using Trizol and 1 μg of total RNA was used to synthesize cDNA using the PrimeScript RT Master Mix kit (Takara, RR036A), cDNA was subjected to quantitative PCR using the TB Green Premix EX II kit (Takara, RR820A). The primers used in this study were listed in Supplementary Table 2

Immunohistochemical Staining

Human melanoma tissue microarrays (TMAs) were obtained from Bioaitech (K063Me01; Xi'an, China). The microarrays included 38 primary malignant melanomas, 10 lymph node metastatic malignant melanomas, 7 normal skin tissues and 6 normal tissues from relevant metastatic sites. All the tissue samples of TMAs are from different individuals. The analysis of tissue sections was conducted using immunohistochemical (IHC) staining. We performed IHC staining on the same paraffin-embedded tissue blocks that were used for clinical diagnosis. The following antibodies were used: USP10, FOXM1, Ki67, CD31 and Caspase-3.

Immunoprecipitation

For immunoprecipitation assays, the protease inhibitor cocktail is added to 1% NP40 lysate and the cells are lysed at 4°C for one hour. The lysate was centrifuged at 4°C and 12,000 rpm for 10 minutes, a portion of the whole cell lysate was used as input, and the rest of the lysate was incubated overnight at 4°C with the appropriate antibody and protein A/G agarose beads (Santa Cruz). At the end of incubation, the immunoprecipitation complexes were washed three times with 200 μL PBS. Whole cell lysate samples and immunocomplex samples were analyzed by Western blotting.

Protein purification and GST Pull-Down assay

Plasmids encoding GST-Vector and GST-USP10 were transformed into BL21 DE3-competent cells and then induced with 1 mM IPTG (I8070, Solarbio, China) for 5 hours. Subsequently, E. coli BL21 (DE3) cells were disrupted via ultrasound, and the supernatant was collected after centrifugation. The supernatant was then incubated with Glutathione Sepharose 4B (17-0756-01, GE Healthcare, USA) at 4°C for 4 hours, followed by thorough washing with PBS. Flag-FOXM1 protein from transfected 293T cells was enriched using agarose beads, and the protein was eluted from the beads using Flag-peptide (F4799, Sigma-Aldrich). The Flag-FOXM1 protein was then incubated with Glutathione Sepharose 4B overnight at 4°C. Precipitates underwent five washes with PBS, followed by analysis using SDS-PAGE and subsequent Western blotting analysis.

FOXM1 half-life and deubiquitination assay

For half-life assay, the indicated melanoma cells were treated with or without Cycloheximide(10μg/ml). Cell lysates were analyzed with the indicated antibodies.
For FOXM1 deubiquitination assay, the HA-Ub plasmid was transfected into USP10 knockdown or overexpressing cells. Cells were treated with MG132 for 6 hours and then collected for immunoprecipitation. Subsequently, cells were incubated with FOXM1 antibody for 12 hours at 4°C along with cell whole lysates and protein A/G agarose beads (Santa Cruz). At the end of the incubation the agarose beads were washed three times with 200 μl PBS buffer and boiled at 100°C for 6 minutes. This was followed by analysis using Western blotting.

In *vitro* deubiquitination assay

Flag-FOXM1 and HA-Ub were co-transfected into 293T cells. The ubiquitinated Flag-FOXM1 was then precipitated from the cell lysate using a Flag antibody. GST and GST-USP10 were transformed into E. coli BL21 (DE3) and induced with IPTG for 5 hours before being ultrasonically broken. The GST and GST-USP10 proteins were then purified using Glutathione Sepharose 4B (17-0756-01, GE Healthcare, USA) and eluted with 20 mM Glutathione. Finally, GST-USP10 was incubated with ubiquitinated Flag-FOXM1 in a deubiquitination buffer (20mM Tris-HCl (Ph=8.0), 50mM NaCl, 1mM EDTA, 1mM DTT, 5% Glycerinum) at 37 °C for 3 hours, with or

without Golvatinib. The level of ubiquitination of Flag-FOXM1 in the immunocomplex samples were analyzed by Western blotting.

Promoter reporters and dual-luciferase assay

The wild-type and mutant promoters were constructed into the pGL3basic vector, following which the vector was used to transfect cells. Subsequently, cells were assayed for luciferase activity using the Dual Luciferase Reporter Gene Assay Kit (iGeneBio, LF001) in accordance with the instructions provided by the reagent supplier. The relative quantification of firefly luciferase activity was analysed using Renilla luciferase activity as a benchmark.

EdU assay

EdU detection was accomplished through the utilisation of the EdU-555 Cell Proliferation Detection Kit (Beyotime, C0075S, Shanghai). The cells were observed and documented through the utilisation of a fluorescence microscope.

Senescence-associated β-galactosidase (SA-β-Gal) staining

Cells were inoculated in 12-well plates and subsequently stained 24 hours later using the Cell Senescence β-Galactosidase Staining Kit (Beyotime, C0602, Shanghai). The cells were washed twice with PBS, the staining fixative was fixed at room temperature for 15 minutes, and they were then washed three times with PBS. They were subsequently incubated with the newly configured staining solution at 37°C in the absence of 5% CO_2 for 24 hours. The SA-β-Gal-positive cells (blue) were observed with an inverted light microscope. In the case of cells that require drug treatment, the initial step involved inoculation of the cells in 12-well plates, followed by a 24-hour period of drug treatment prior to the commencement of the SA-β-Gal activity assay.

Cellular Thermal Shift Assay, CETSA

The A375 cells were lysed in NP-40 buffer (1% protease inhibitor cocktail) and then treated with or without Golvatinib for 4 hours at room temperature. The lysates were were divided into six PCR tubes and heated for 5 minutes at 40, 45, 50, 55 and 60 °C. After centrifugation (12,000 rpm, 15 min, 4 °C), the super-natant was collected and detected by Western blotting.

Molecular docking

The USP10 protein(UniProt ID: Q14694) crystal full-length structure of the USP10 predicted by D-I-TASSER, and using COACH to predict ligand binding sites and structure transformed in PDB format. The Drugbank drugs were downloaded from (https://go.drugbank.com/) using the R package (webchem) to obtain The SMILES of the drug was obtained via the PubChem Compound CID, after which the corresponding 3D chemical structure (sdf format file) was downloaded from (https://pubchem.ncbi.nlm.nih.gov) based on the CID. The sdf format file was converted to mol2 format file using obabel tool. Finally, the ligand structure was processed using the prepare_ligand4.py script in MGLTools to generate a pdbqt file for docking. The activity pockets of the target proteins were predicted using P2Rank software. Molecular docking was implemented using the AutoDock Vina software package, using the Vina force field, which defines the centre and dimensions of the grid space according to the pockets, and generates nine ligand poses for each pocket, which were ranked according to their affinity scores in kcal/mol.Autodock Vina wrote the PDBQT output files, which were displayed in ChimeraX using the interactive ViewDockX tool.

Reference

1. Ge Z, Leighton JS, Wang Y, Peng X, Chen Z, Chen H*, et al.* Integrated Genomic Analysis of the Ubiquitin Pathway across Cancer Types. *Cell reports* 2018, **23**(1)**:** 213-226 e213.

2. Liu J, Lichtenberg T, Hoadley KA, Poisson LM, Lazar AJ, Cherniack AD*, et al.* An Integrated TCGA Pan-Cancer Clinical Data Resource to Drive High-Quality Survival Outcome Analytics. *Cell* 2018, **173**(2)**:** 400-416 e411.

3. Wyles SP, Yu GT, Ganier C, Tchkonia T, Lynch MD, Kuchel GA*, et al.* SenSkin: a human skin-specific cellular senescence gene set. *GeroScience* 2025, **47**(3)**:** 2631-2638.

4. Saul D, Kosinsky RL, Atkinson EJ, Doolittle ML, Zhang X, LeBrasseur NK*, et al.* A new gene set identifies senescent cells and predicts senescence-associated pathways across tissues. *Nature communications* 2022, **13**(1)**:** 4827.

5. Qu Y, Ji B, Dong R, Gu L, Chan C, Xie J*, et al.* Single-cell and spatial detection of senescent cells using DeepScence. *Cell genomics* 2025, **5**(12)**:** 101035.
